# Cannabinoid Receptors Are Overexpressed in CLL but of Limited Potential for Therapeutic Exploitation

**DOI:** 10.1371/journal.pone.0156693

**Published:** 2016-06-01

**Authors:** Patricia Freund, Edit A. Porpaczy, Trang Le, Michaela Gruber, Clemens Pausz, Philipp Staber, Ulrich Jäger, Katrina Vanura

**Affiliations:** Division of Hematology and Hemostaseology, Department of Medicine I, Medical University of Vienna, Vienna, Austria; Complutense University, SPAIN

## Abstract

The cannabinoid receptors 1 and 2 (CNR1&2) are overexpressed in a variety of malignant diseases and cannabinoids can have noteworthy impact on tumor cell viability and tumor growth. Patients diagnosed with chronic lymphocytic leukemia (CLL) present with very heterogeneous disease characteristics translating into highly differential risk properties. To meet the urgent need for refinement in risk stratification at diagnosis and the search for novel therapies we studied CNR expression and response to cannabinoid treatment in CLL. Expression levels of CNR1&2 were determined in 107 CLL patients by real-time PCR and analyzed with regard to prognostic markers and survival. Cell viability of primary CLL cells was determined in suspension and co-culture after incubation in increasing cannabinoid concentrations under normal and reduced serum conditions and in combination with fludarabine. Impact of cannabinoids on migration of CLL cells towards CXCL12 was determined in transwell plates. We found CNR1&2 to be overexpressed in CLL compared to healthy B-cells. Discriminating between high and low expressing subgroups, only high CNR1 expression was associated with two established high risk markers and conferred significantly shorter overall and treatment free survival. Viability of CLL primary cells was reduced in a dose dependent fashion upon incubation with cannabinoids, however, healthy cells were similarly affected. Under serum reduced conditions, no significant differences were observed within suspension and co-culture, respectively, however, the feeder layer contributed significantly to the survival of CLL cells compared to suspension culture conditions. No significant differences were observed when treating CLL cells with cannabinoids in combination with fludarabine. Interestingly, biologic activity of cannabinoids was independent of both CNR1&2 expression. Finally, we did not observe an inhibition of CXCL12-induced migration by cannabinoids. In contrast to other tumor entities, our data suggest a limited usability of cannabinoids for CLL therapy. Nonetheless, we could define CNR1 mRNA expression as novel prognostic marker.

## Introduction

Cannabinoids, the active components of the hemp plant *Cannabis sativa*, have been used for centuries for medical and recreational purposes. These compounds exert their activity by binding the cannabinoid receptors.

Cannabinoid receptors 1 and 2 (CNR1/CNR2; CB1/2) belong to the group of G protein-coupled receptors (GPR) and are part of the endocannabinoid system. The native ligands of the two receptors, such as 2-arachidonoyl glycerol, are produced on demand and fulfill a variety of functions. Although other receptors have been described for the endocannabinoid system [1], CB1&2 still are the two receptors for which most knowledge has been gathered.

CNR1 is primarily expressed in the brain, CNR2 in cells and tissues of the immune system, but both receptors have also been found outside these main sites of expression [2–4]. The two receptors appear to mediate similar responses and to exert overlapping influences, they seem to interact with respect to inflammatory and neurologic/psychotic conditions [5–9], and are involved in migration of cells of different origin under different physiological states [10–12]. Thus, 2-arachidonoyl glycerol acts as chemo-attractant for both immature and mature B-cells via CB2 [13–16] and appears to interfere with CXCR4 expression and/or CXCL12 induced migration [14]. Similar migratory effects were reported also for other cannabinoids [17], although the extent of these effects seems to be variable [18, 19].

Overexpression of both receptors has been found both in solid tumors and hematologic malignancies and cannabinoids were shown to inhibit cell migration, angiogenesis, to reduce proliferation and viability, to induce apoptosis *in vitro* and reduce tumor burden *in vivo* [4, 20–32]. The sensitivity of mantle cell lymphoma (MCL), chronic lymphocytic leukemia (CLL), and Hodgkin lymphoma (HL) cell lines to cannabinoids was linked to the overexpression of CNR1 and/or CNR2 [23, 33, 34]. While some of these reports used relatively selective agonists like ACEA (CB1), JWH133, or JWH015 (both CB2) [25, 30, 35–37], in the majority of studies cannabinoids were tested which appear to display broader activity on G-coupled protein receptors [23, 28, 31, 33, 38]. Thus cannabidiol acts as CB1 antagonist, CB2 inverse agonist, GPR55 antagonist, and agonist for the VR1 vanilloid and the μ-opioid receptors [39, 40], (R)-(+)-methanandamide as CB1 agonist but also displays activity at vanilloid receptors and other G-protein coupled receptors and ion channels [1, 41].

In solid tumors, expression of the two cannabinoid receptors has been linked to patient outcome. In hepatocellular carcinoma and mobile tongue squamous cell carcinoma, both CB1 and CB2 overexpression was associated with good prognosis [42, 43]. In contrast, CB1 expression was reported to be a marker of bad prognosis in prostate and colorectal cancer [44–46], while CB2 was shown to be a poor prognostic marker in colon cancer [32] and was linked to poorer survival in HER2 positive breast cancer and squamous cell carcinoma of head and neck [47, 48]. Whether increased expression of one of the two receptors or both has clinical implications in hematologic malignancies appears to be variable [49–51].

The development of targeting drugs in recent years has greatly improved therapeutic options in CLL [52–54]. However, it is not known how long targeting molecules will display their potential before patients develop resistances and/or progress. In this line, several reports already discussed genetic changes developing during treatment with such compounds [55, 56]. CLL, like other malignancies, consists of a pool of malignant clones [57–59], which develop and evolve during disease course. Changes in this clonal landscape may occur during treatment and/or due to the acquisition of resistance mutations. Therefore, there still is an urgent need for agents which can be used for combination therapy as well as supportive regimens to increase treatment options and to improve patient care.

Based on the reported aberrant expression of cannabinoid receptors in neoplasms, we studied the expression of the two receptors in CLL patients analyzing it in relation to clinical parameters to determine their usability for prognosis. Additionally, considering the versatile aspects of cannabinoid actions, we evaluated the potential of cannabinoids for use in CLL therapy.

## Materials and Methods

### Patient material

Peripheral blood samples were collected from 107 consecutive patients diagnosed with CLL at the Division of Hematology and Hemostaseology of the General Hospital in Vienna, Austria. All patients and the four healthy volunteers included in the study signed informed consent according to the Declaration of Helsinki. The study was approved by the Ethics Committee of the Medical University of Vienna (approval Nr: 1011/2012). Clinical characteristics for the patients used in mRNA expression analysis are listed in S1 Table.

Peripheral blood mononuclear cells (PBMC) were isolated by Ficoll (Biocoll, Biochrom, Berlin, Germany) separation following standard procedures, cells were stored viable in liquid nitrogen. For protein analyses, 1x10^7^ PBMCs were centrifuged, the supernatant was discarded and cell pellets were stored at -80°C. For RNA extraction, primary cells were stored at -80°C in TRIzol (Life Technologies Ltd, Paisley, UK). B lymphocytes from healthy donors were isolated using the EasySep^™^ Human B Cell Enrichment Kit without CD43 Depletion (STEMCELL Technologies SARL, Grenoble, France) following the manufacturer´s protocol.

### Reagents

Cannabinoids used in cytotoxicity and migration experiments were ACEA (CB1 agonist), AM251 (CB1 antagonist, μ-opioid receptor antagonist, GPR55 agonist, ion channel activation), AM630 (CB2 antagonist/inverse agonist, weak partial CB1 agonist, ion channel activation), (-)-cannabidiol (CNB) (GPR55 & weak CB1 antagonist, CB2 inverse agonist, weak agonist at VR1 vanilloid receptors, and modulator at opioid receptors), JWH133 (CB2 antagonist), and R-(+)-methanandamide (RM) (CB1 agonist, activity against GPR and ion channels) purchased from TOCRIS Bioscience (Bristol, UK). Fludarabine (2-Fluoroadenine-9-β-D-arabinofuranoside, SIGMA-ALDRICH, Vienna, Austria) served as control in cytotoxicity experiments, CXCL12/SDF-1α (R&D SYSTEMS, Abingdon, UK) and CXCR4 antagonist AMD3100 octahydrochloride (TOCRIS Bioscience, Bristol, UK) were controls in migration experiments.

### Primary antibodies

The following cannabinoid receptor directed antibodies were tested:

Anti-CB1 antibodies: Cat.No. PA1-745, Thermo Fisher Scientific Inc., Waltham, MA, USA; Cat.No. GTX100517, GeneTex Inc., Irvine, CA, USA; Cat.No. AF1185a, Abgent, San Diego, CA, USA.

Anti-CB2 antibodies: Cat.No. AF1575a, monoclonal, Abgent, San Diego, CA, USA; Cat.No. AF1186a, polyclonal, Abgent, San Diego, CA, USA; Cat.No. PA1-744, Thermo Fisher Scientific Inc., Waltham, MA, USA; Cat.No. GTX100391, GeneTex Inc., Irvine, CA, USA.

Cat.No. CB1001, anti-GAPDH monoclonal antibody, Calbiochem/EMD Millipore, Merck, Darmstadt, Germany.

Recombinant proteins for Western blots: CB1 and CB2 human recombinant proteins (Cat.Nos. H00001268-G01 and H00001269-G01; Abnova, Taipei City, Taiwan).

### Secondary antibodies

IRDye 680 conjugated goat anti-mouse polyclonal IgG (H+L) (Cat.No. 926–32220), and IRDye 800CW conjugated goat anti-rabbit polyclonal IgG (H+L) (Cat.No. 926–32211), both purchased from LI-COR (Bad Homburg, Germany).

### RNA extraction and cDNA synthesis

RNA was extracted from samples of CLL patients and healthy donors using TRIzol, RNA was dissolved in 10 μl DEPC water, and the amount of isolated RNA measured. Two μg of RNA were used for cDNA synthesis (all products from Promega Corporation, Madison, USA), cDNA was stored at -20°C until real time PCR.

### Real time PCR

Real time PCR was carried out using TaqMan Gene Expression Assays on demand for Cannabinoid receptors 1 and 2 (Cat.Nos. Hs00275634_m1 and Hs00361490_m1, Life Technologies Ltd, Paisley, UK) according to the manufacturer´s protocol. Applied Biosystems^®^ Human ACTB (Cat.No. 4326315E) served as housekeeping gene. Samples were run in duplicates on an ABI Prism 7000 Sequence Detector and analyzed using the SDS Software. For calculation of mRNA expression, the ΔΔCt-method was used [60], for which CD19 sorted pooled healthy B cells were set as 1.

### Protein isolation and Western Blot

Proteins from patient samples were prepared using RIPA Buffer, concentration was determined using Pierce BCA Protein Assay Kit (Thermo Fisher Scientific Inc., Rockford, USA). Sixty μg of protein per lane were separated by 10% SDS PAGE and transferred to PVDF Immobilon-FL Transfermembrane (pore size 0.45 μM; Merck Millipore, Billerica, US). Membranes were incubated for 16 hours at 4°C with the primary antibody, were washed 3 times for 5 minutes with PBS (PAA laboratories, Pasching, Austria) + 0.1% Tween20 (Sigma Aldrich, St. Louis, MO, USA), and subsequently incubated 45 minutes at room temperature (RT) in the dark with the secondary antibody before detection on an Odyssey Imager (LI-COR, Bad Homburg, Germany)

### Cell culture

All cells were cultured under standard conditions (95% humidity, 5% CO_2_, 37°C). M2-10B4 (purchased from the American Type Culture Collection; http://www.lgcstandards-atcc.org) were kept in RPMI1640 containing 10% fetal calve serum (FCS) and 1% Penicillin/Streptomycin (PS) as were primary CLL cells and healthy PBMC. All reagents were purchased from Life Technologies (Carlsbad, CA, USA).

### Drug incubations and viability tests

All experiments were performed using medium without phenol red. Viability was determined using the CellTiter-Blue^®^ Viability Assay (Promega, Madison, WI, USA) following the manual. Experiments with M2-10B4 were done in triplicates and repeated twice. Drug incubations with primary cells were done in triplicates using 10–18 samples from CLL patients and 2–3 healthy donors (see respective figures for the number of samples included in each incubation). Concentrations ranged between 0 and 100 μM, the optimal range having been determined in preliminary experiments. Concentrations are indicated for each compound and experiment in the respective figures. Cells incubated with vehicle only served as controls, vehicle did not exceed 1% of experimental volume. Viability was calculated as the mean of all replicates and was normalized to vehicle control (set as 100%).

Primary cells from both CLL patients and healthy donors were tested in suspension culture for 48 h at 37°C at concentrations of 3x10^6^ cells/ml in 96 well plates before viability was measured. For co-culture incubations of CLL cells, 2x10^5^ M2-10B4 cells were seeded in 12 well plates and incubated for 24 h under standard conditions. On the next day, primary CLL cells were transferred to the wells at a concentration of 3x10^6^ cells/ml. Compounds were added and plates were incubated for 48 h at 37°C. Then, CLL cells were transferred to 96 well plates before carrying out the CellTiter Blue assay.

To determine whether CB1&2 agonists and antagonists exert an additive cytotoxic effect when combined with fludarabine, CLL primary cells (n = 5) were prepared for co-culture incubation as described above. Cells were treated with increasing concentrations of ACEA and JWH133, and single concentrations of AM251, AM630, and CNB. Using the same setup, cells were pre-incubated with cannabinoids for 30 minutes before 5 μM fludarabine was added. For comparison, primary cells were incubated in co-culture with vehicle alone, fludarabine (5 μM) alone, AMD3100 (0,629 mM) alone, and were pre-treated with AMD3100 for 30 minutes before fludarabine was added. Plates were left for 48 h at 37°C before viability was determined.

To evaluate a potential effect of serum on cannabinoid toxicity, primary CLL cells from 5 patients were tested at different FCS concentrations (1, 2.5, 5, and 10%) both in suspension and in co-culture with M2-10B4. Preparation of experiments and determination of viable cells were done as described above.

### Migration assays

CLL cells of 7 patients were sorted using the Human B cell Enrichment Kit (CatNo.8804-6818-74, Affymetrix eBiosciences, San Diego, CA, USA) following the manufacturer´s recommendations. Resulting CD19+CD5+ cell purity was 97.7% ± 1.04. Cells (5x10^6^ cells/ml) were incubated with AM251, AM630, JWH133, ACEA, or with a combination of antagonist + agonist. Incubations with vehicle, with AMD3100, and without CXCL12 served as controls. Compound concentrations were chosen to exert minimal cytotoxic effects on cells. A list of concentrations and combinations can be found in S2 Table.

Six hundred μl RPMI1640 without phenol red were pipetted into the bottom chambers of 24-well polycarbonate membrane transwell plates (6.5 mm diameter, 5.0 μm pore size; Corning Inc., Corning, NY, USA), CXCL12 (0,1 μg/ml) or vehicle (0.1% PBS with 0.1% BSA) was added to the medium. After addition of transwell inserts, 100 μl of treated cells were transferred to the upper chamber. Migration was allowed to occur for 4h at 37°C. The contents of both the upper and the bottom wells were transferred to separate tubes, centrifuged, pellets were resuspended in PBS, and cells were counted by FACS on a BD FacsCanto II Flow Cytometer.

A similar set of experiments was done with unsorted PBMC from CLL patients (N = 5) for which cells were pre-incubated with agonist, antagonist, or the combination of both before being transferred to the transwell plates. Incubation time was 0.5 h for antagonist, 1 h for agonist, and 1.5 h for antagonist plus agonist. An overview of these combinations can also be found in S2 Table. In this setting, cells were counted in a Neubauer counting chamber.

Migration is shown as means + SD of the migration index which was calculated as the number of cells migrated in the presence of compound divided by the number of cells migrated in the presence of vehicle [61–63].

### Statistical methods

CNR1 and 2 expressions are given as median, quartiles and range. The median was set as cut-off for CNR expression to distinguish CNR high and low expression groups. The impact of CNR mRNA expression on survival was tested by Kaplan-Meier plots (*P* values from log-ranks tests) and quantified by hazard-ratios in univariate and multivariable Cox regression analyses. Progression free survival was calculated from first treatment to progression, overall survival was measured from date of first diagnosis to follow up and treatment free survival was calculated from date of first diagnose till first treatment or follow up. Prognostic markers were compared between groups using Chi^2^-tests. Pairwise comparison of migration experiments and viability assays was done by t-test. For this, IC_50_ values were compared between CLL suspension and co-culture at the same compound concentration, between CLL suspension culture and healthy donors in suspension at the same compound concentration, and—for reduced serum conditions—between CLL suspension and co-culture at the same compound and serum concentrations. In addition, again for reduced serum conditions, IC_50_ values were compared within CLL suspension and co-culture experiments, respectively, at the same compound but at different serum concentrations. Pearson correlation and linear regression analyses were performed to determine a potential association between IC_50_ values and CNR mRNA expression. P-values ≤ 0,05 were considered statistical significant. Computations were performed using SPSS or GraphPad Prism.

## Results

### CNR mRNA and protein expression in CLL

CNR1 mRNA expression ranged from 0.00 to 140.39 with a median expression of 1.52. CNR2 mRNA had a median expression of 3.77, ranging from 0.06 to 26.54. Receptor expression of healthy, CD19 sorted B-cells was set as 1.

Using the median expression as cut-off, patients were split into high and low expressing groups for both receptors. Medians (ranges) were 0.23 (0.00–1.41) and 7.16 (1.52–140.39) for the CNR1 low and high expressing groups, and 2.35 (0.06–3.72) vs. 5.41 (3.77–26.54) for CNR2 low and high expressing groups, respectively. The number of patients in the 4 groups were 31 in the double low expressing group, 44 in the mixed group (one receptor high, the other low expressing), and 32 were double high expressers.

In order to determine whether receptor protein expression follows mRNA expression, we carried out Western blots using different commercially available antibodies (listed in Methods). In these experiments, we included recombinant proteins for both receptors as positive controls for CB1&2 expression. Despite testing different protein extraction and transfer protocols, we were not able to a) determine specific bands, and b) detect differences in receptor expression (data not shown). We concluded that the antibodies used in our experiments were unspecific, most likely showing crossreactivity with both receptors and/or detecting other proteins concomitantly. Subsequently, protein data were excluded from any analysis.

### CNR1 but not CNR2 mRNA is a prognostic marker in CLL

**Fig 1:**
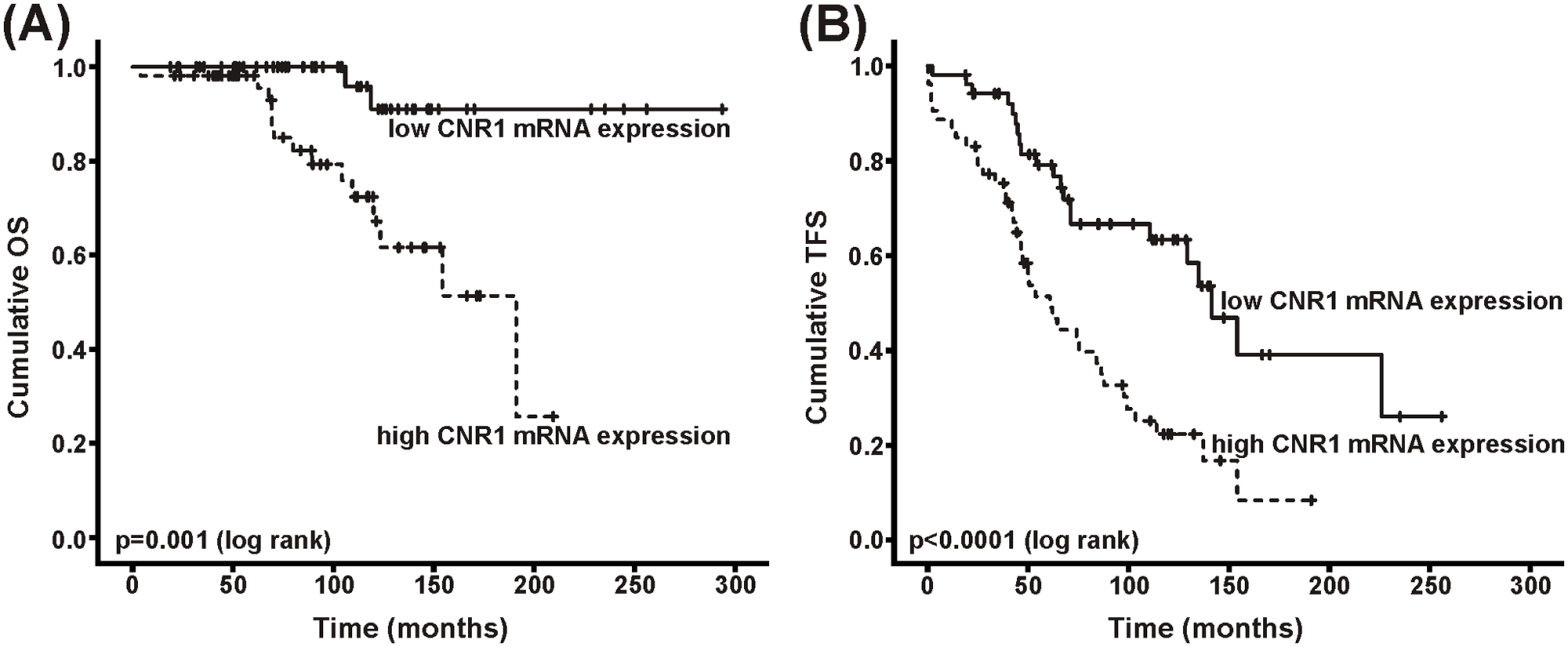
High CNR1 mRNA expression (≥ 1.52) confers significantly shorter survival in CLL patients (n = 107). (A) High expressing patients had a mean overall survival (OS) of 153 months compared to 277 months in low expressing patients (p = 0.001). (B) The mean treatment free survival (TFS) was 75 months in the CNR1 high group vs. 150 months in the CNR1 low group (p<0.0001).

Next, we analyzed patient data with regard to high and low mRNA expression of CNR1 and CNR2, respectively. We found that, based on the level of CNR1 expression, patient characteristics were significantly different for two prognostic markers. Thus, persons displaying high CNR1 mRNA expression were more likely to have unmutated IgHV genes (54.3 vs. 32.7%; p = 0.033), or high CD38 expression (42.6 vs. 21.6%; p = 0.026) (Table 1). In addition, CNR1 high expressing patients had a significantly shorter overall and treatment free survival (OS; TFS) (OS: HR = 8.615; 95% CI 1.947–38.112; p = 0.001; TFS: HR = 2.770; 95% CI 1.603–4.785; p<0.0001; Fig 1). No such difference could be observed for progression free survival (PFS; p = 0.171 (log rank)). Of note, CNR mRNA expression did not correlate with the fraction of CD19+CD5+ cells. In multivariate analyses, the influence of CNR1 mRNA expression on OS was independent from CD38 expression, IgHV mutational status, clinical stage B & C, and lymphocyte doubling time, but not so for TFS.

**Table 1:** Comparison of patient characteristics between CNR1 high and low mRNA expressing groups.

	CNR1 low	CNR1 high	p-value
**Age at diagnosis** [years]			
Median (Range)	59 (25–85)	63 (39–82)	
**Sex** [%]			
Female:Male	47.2:52.8	38.9:61.1	
**CD19+CD5+ cells** [%]			
Median (Range)	79.0 (28–97)	86.0 (42–97)	
Mutational status* [%]			
Unmutated	32.7	54.3	**0.033**
Mutated	67.3	45.7	
**CD38** [%]			
Low < 30	78.4	57.4	**0.026**
High ≥ 30	21.6	42.6	
Median (Range)	5.0 (0–91)	23.0 (0–85)	
**Binet at diagnosis** [%]			
A	88.5	81.1	0.296
B/C	11.5	18.9	
**Lymphocyte doubling time** [%]			
Low < 1 year	18.0	30.4	0.154
High ≥ 1 year	82.0	69.6	
**Del13q** [%]			
Unmutated < 5.0	46.0	50.0	0.686
Mutated ≥ 5.0	54.0	50.0	
**Del11q** [%]			
Unmutated < 8.6	84.0	71.2	0.121
Mutated ≥ 8.6	16.0	28.8	
**Del17p** [%]			
Unmutated < 10.2	90.0	92.3	0.681
Mutated ≥ 10.2	10.0	7.7	
**Tris12** [%]			
Unmutated < 3.7	86.0	92.3	0.305
Mutated ≥ 3.7	14.0	7.7	
**Rearr14q** [%]			
Unmutated < 3.0	91.8	81.6	0.136
Mutated ≥ 3.0	8.2	18.4	

*Cut-off 98% germline homology. Abbreviations: Del, deletion; Tris, trisomy; Rearr, rearrangement. P-values (Pearson Chi-Square) reaching statistical significance are bold.

In contrast, no significant differences could be observed between CNR2 high and low expression groups. CNR2 mRNA expression levels were not associated with any of the established prognostic markers, nor did high expression translate into shorter OS, TFS, or PFS (p = 0.763, p = 0.229, p = 0.089, respectively (log rank)). Patient characteristics in relation to CNR2 high and low expression are listed in S3 Table, Kaplan Meier Analyses for OS and TFS are shown in S1 Fig.

### Impact of cannabinoids on viability of primary cells

**Fig 2:**
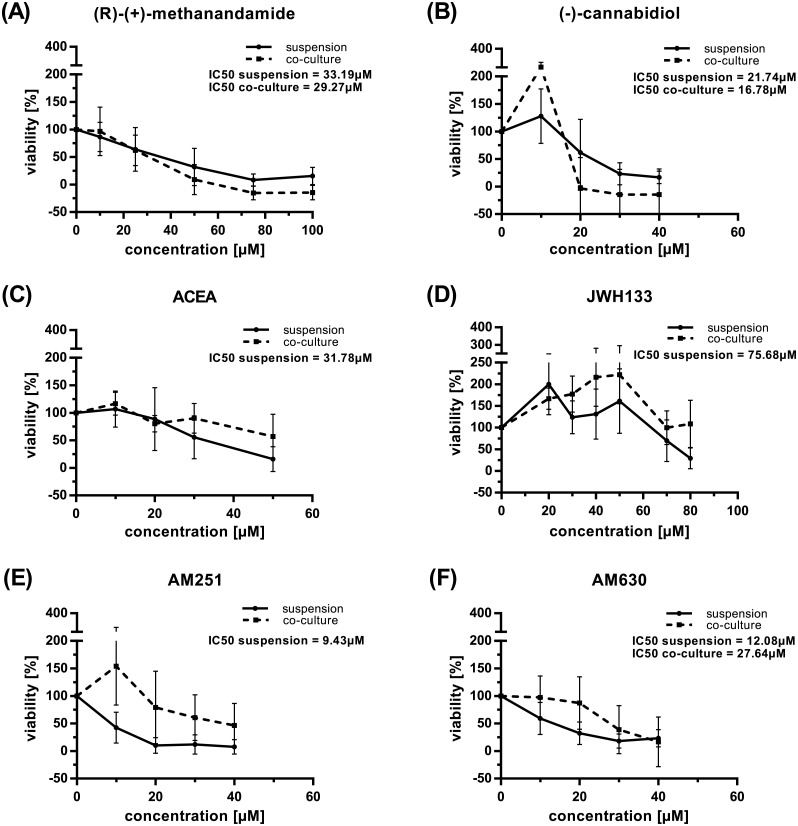
Cytotoxic impact of cannabinoids on CLL primary cells. PBMC from CLL patients were incubated in triplicates both in suspension culture and in co-culture with M2-10B4 mouse fibroblast cells in increasing concentrations of compounds. Viability was determined after 48h, mean values and standard deviations are shown. (A) (R)-(+)-methanandamide (N = 10). (B) (-)-cannabidiol (N = 18). (C) ACEA (N = 16). (D) JWH133 (N = 16). (E) AM251 (N = 16). (F) AM630 (N = 16). For ACEA, JWH133, and AM251, the 50% reduction in viability required for IC_50_ calculation could not be reached in co-culture. Note different scale on x-axis in A and D.

A dose dependent reduction of viability could be observed in CLL primary cells with increasing concentration of drug both in suspension and co-culture experiments (Fig 2).

**Fig 3:**
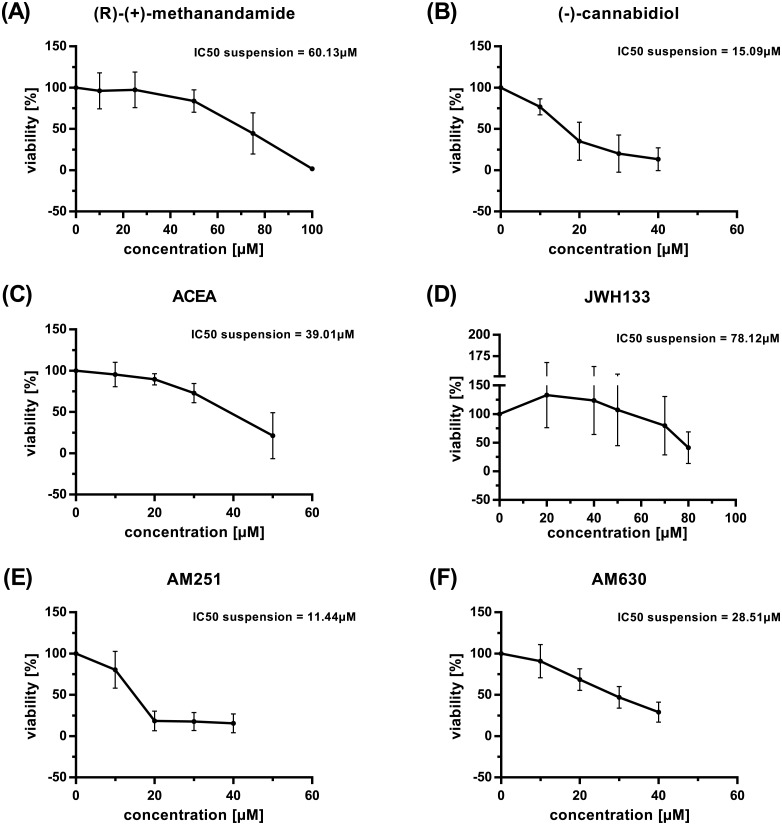
Cytotoxic impact of cannabinoids on primary cells from healthy individuals. PBMC from 3 healthy donors were incubated in triplicates in suspension culture in increasing concentrations of compounds. Viability was determined after 48h, mean values and standard deviations are shown. (A) (R)-(+)-methanandamide. (B) (-)-cannabidiol. (C) ACEA. (D) JWH133. (E) AM251. (F) AM630. Note different scale on x-axis in A and D, note different scale on y-axis in D.

This reduction was more pronounced in suspension culture compared to co-culture for the selective CB1 antagonist AM251 (p = 0.0431) compared to the selective CB1 agonist ACEA (p = 0.1855) and more pronounced for the selective CB2 agonist JWH133 (p = 0.0527) compared to the selective CB2 antagonist/inverse agonist AM630 (p = 0.1353). No differences between the two culture conditions could be observed for (R)-(+)-methanandamide (RM) (CB1 agonist, activity against vanilloid and other G-protein coupled receptors and ion channels) and (-)-cannabidiol (CNB) (weak CB1 antagonist, CB2 inverse agonist, interacting also with GPR55, vanilloid receptors and opioid receptors) (p = 0.1464 and p = 0.6549, respectively). These data suggest a protective effect of the fibroblasts in incubations with AM251 and JWH133. The loss of this effect, at least for RM, CNB, and AM630, may be explained by the cytotoxicity these compounds exerted on M2-10B4 fibroblast cells alone, IC_50_ values being in the range of CLL primary cells (Table 2). A dose dependent reduction of viability was also observed for PBMC of healthy individuals in suspension (Fig 3), IC_50_ values were in the same range as those of primary CLL cells under the same conditions except for RM and AM630 for which IC_50_ values were approximately twice as high (Table 2).

**Table 2:** Impact of cannabinoids on the viability of primary cells and mouse fibroblasts.

	RM	CNB	ACEA	JWH133	AM251	AM630
CLL suspension	33.19	21.74	31.78	75.68	9.43	12.08
CLL co-culture	29.27	16.78	NR	NR	NR	27.64
HD suspension	60.13	15.09	39.01	78.12	11.44	28.51
M2-10B4	34.55	13.52	NR	NR	NR	28.27

IC_50_ values (μM) are based on the mean values of 10 to 18 CLL PBMC and 3 HD PBMC which were incubated in triplicates in a concentration range of 0 to 100 μM of drugs for 48h. Viability of CLL cells was assessed both in suspension culture and in co-culture with the mouse fibroblast cell line M2-10B4. Healthy donor (HD) cells were incubated in suspension culture only. Abbreviations: RM, (R)-(+)-methanandamide; CNB, (-)-cannabidiol; NR, IC_50_ not reached.

Cannabinoids reportedly interfere with cell-cell crosstalk which may potentially affect the therapeutic efficacy of drugs. Thus, we combined cannabinoids with fludarabine to explore potential synergies between tested drugs. Pre-incubation with cannabinoids before the addition of fludarabine generally led to a reduction in cell viability compared to cannabinoids alone (N = 5) (S2 Fig). These differences, however, were not statistically significant. Exception was the CB1 agonist ACEA where the concentration dependent cytotoxicity increased reaching statistical significance in the 40 μM incubations (10 μM ACEA vs. 10 μM ACEA+Flu p = 0.117; 20 μM ACEA vs. 20 μM ACEA+Flu p = 0.076; 40 μM ACEA vs. 40 μM ACEA+Flu p = 0.047). Impact of cannabinoids and combinations were not statistically significant either when compared to controls (N = 6) with fludarabine alone, with AMD3100 alone or with AMD3100+Fludarabine (S2 Fig).

Serum factors may interact with cannabinoids resulting in a reduction in biologic drug activity. To investigate such an effect, primary cells were incubated with increasing concentrations of cannabinoids under reduced serum conditions, both in suspension and in co-culture (N = 5). Although not statistically significant within culture conditions, the results suggest that reduced serum concentrations added to the cytotoxic effect of the compounds, particularly at lower drug concentrations (1 and 2.5% vs. 5 and 10% FCS; S3 Fig), while this difference leveled out at higher compound concentrations. A pairwise comparison of corresponding compound concentrations between the two culture conditions (Table 3) showed a significant advantage for CLL cells in co-culture at 1 and 2.5% serum concentrations and in most cases also at 5 and 10% serum concentrations, again underlining the importance of cell-cell interaction for CLL cell survival especially at low serum conditions. Once more CNB appeared to be an exception probably due to its impact on the feeder cells (Tables 2 and 3).

**Table 3:** P-values of the pairwise comparison of IC_50_ values between suspension and co-culture in serum reduced experiments.

	RM	CNB	ACEA	JWH133	AM251	AM630
1% serum	**0.0336**	0.125	**0.0311**	**0.0278**	**0.03**	**0.0278**
2.5% serum	0.7254	**0.03**	**0.0311**	**0.0412**	**0.0396**	**0.0468**
5% serum	**0.0154**	0.3255	**0.0279**	**0.0282**	0.0823	0.0993
10% serum	**0.05**	0.2511	**0.0468**	**0.0386**	0.1021	**0.0337**

Student´s t-test was applied, p-values reaching statistical significance or borderline significance are bold. Abbreviations: RM, (R)-(+)-methanandamide; CNB, (-)-cannabidiol;

Of note, sensitivity to cannabinoids was not associated with mRNA expression of either of the receptors (S4 and S5 Figs) indicating that the cytotoxic affect exerted by the tested cannabinoids was not or only partially mediated by cannabinoid receptors.

### Migration assays

**Fig 4:**
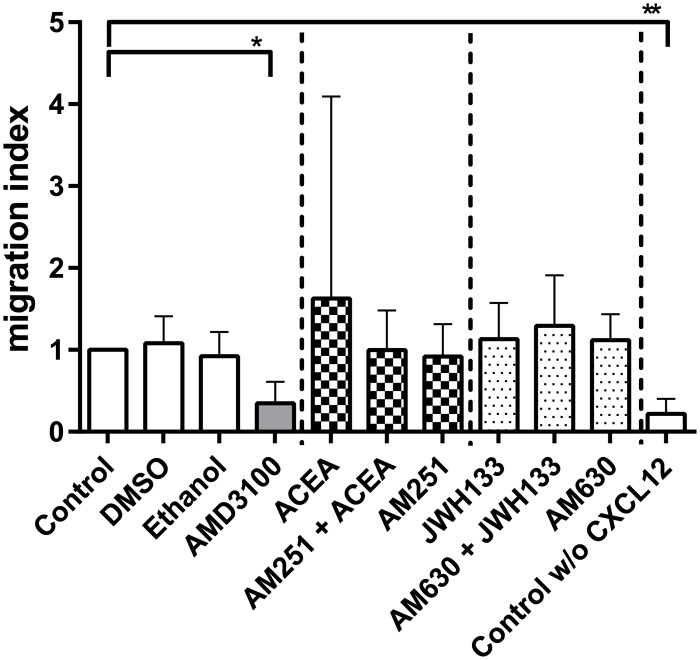
Impact of cannabinoids on CLL cell migration. B-cell enriched primary cells of 7 CLL patients (97.7% ± 1.04 CD19+CD5+) were incubated in transwell plates for 4h before the number of migrated cells was determined. Control experiments included CXCL12 alone (control), no CXCL12 (control w/o CXCL12), incubation with vehicle (DMSO, ethanol), and incubation with the CXCR4 inhibitor AMD3100. CLL cells were incubated either with agonist (ACEA, JWH133), antagonist (AM251, AM630), or a combination of antagonist plus agonist before migration (CB1: AMS251&ACEA; CB2: AM630&JWH133). Bars represent the mean values of migration indices + standard deviations, hatched lines indicate experimental blocks. *p = 0.0006; **p<0.0001.

In T-cells, cannabinoids were shown to inhibit CXCL12 directed migration. Considering the importance of the CXCL12-CXCR4 axis in CLL, we studied whether and to which degree cannabinoids might interfere with CXCL12 mediated CLL cell migration. As shown in Fig 4, vehicle did not induce any significant changes in migration towards CXCL12, in contrast AMD3100 inhibited migration significantly (p = 0.0006) compared to controls. Both CB1&2 agonists (ACEA, JWH133) and antagonists (AM251, AM630) did not significantly influence the migratory behavior of purified CLL cells towards CXCL12. Likewise, combination of antagonist plus corresponding agonist (AM251+ACEA for CB1; AM630+JWH133 for CB2) did not lead to significant changes, either, compared to migration in vehicle containing controls. CLL cells incubated without CXCL12 showed a significantly reduced migratory behavior compared to controls (p<0.0001) (Fig 4).

Experiments with unsorted PBMC from CLL patients using the same compound concentrations but pre-incubating the cells before migration led to similar results, with significantly reduced migration in the absence of CXCL12 (p<0.0001), significant inhibition of migration towards CXCL12 in the presence of AMD3100 (p = 0.0016), and no significant block of migration in incubations with cannabinoids (S6 Fig).

## Discussion

Known for their psychoactive effects, cannabinoids have been used for centuries both for recreational and medical purposes. Upon the discovery of the receptors involved, drugs were developed exploiting the receptors in order to treat accompanying symptoms in various diseases [4, 64, 65]. In addition, cannabinoids displayed a number of anti-tumor effects in solid tumors [4, 20, 22, 24, 26, 27, 29] and in hematologic malignancies [23, 66] which led us to determine the expression of cannabinoid receptors and to evaluate the therapeutic potential of cannabinoids in CLL.

We found that both cannabinoid receptors were overexpressed in CLL cells compared to healthy B-cells. On the mRNA level, CNR2 had a higher median expression than CNR1, which, on the other hand, had a wider range of expression compared to CNR2. When split into high and low expressing groups based on median receptor expression, only CNR1 could be shown to have prognostic value, not CNR2. Although previous publications reported an overexpression of one or both cannabinoid receptors in hematologic malignancies [22, 23, 34, 49, 67], reports regarding a potential role of this overexpression in the clinics are few [49–51]. More information is available for solid tumors, where in most studies either CB1 or CB2 expression was linked to poorer patient outcome [32, 43–48, 68]. Here, we provide for the first time evidence pertaining prognostic relevance of cannabinoid receptors in lymphoid malignancies.

On the protein level, however, we could not detect any differences in expression in high and low expressing patient groups. Although some studies reported the detection of receptor protein levels in relation to mRNA expression [22, 23], we were not able to reproduce this observation. This was likely due to a lack of specificity of the antibodies used in this study. Our data are corroborated by two studies in which several commercially available antibodies were tested for CB1&2, respectively. Both groups reported great differences between the claimed specificity and the observed high degree of unspecific detection of protein by these antibodies [69, 70]. Therefore, cannabinoid receptor protein expression was excluded from further analysis.

We continued by evaluating the two receptors as potential therapeutic targets in CLL. For this, a specific agonist and antagonist pair was chosen for each receptor (CB1: agonist ACEA, antagonist AM251; CB2: agonist JWH133, antagonist/inverse agonist AM630). While these pairs are highly selective for either CB1 or CB2, respectively, a certain degree of promiscuous activity appears to be a common feature to them all. AM251 and AM630 are also ion channel activators [71], AM251 additionally acts as agonist at GPR55 and antagonist at μ-opioid receptors [40, 72]. JHW133 not only acts on CB2 but also the TRPV1 vanilloid receptor plus showed off-target effects in chemotaxis experiments in macrophages [73, 74]. Besides these two ligand pairs, two compounds were included which are being widely used in cytotoxic experiments and which have long been known for broader activity: R-(+)-methanandamide (RM) is CB1 agonist, shows activity at GPR and ion channels [1, 25], and (-)-cannabidiol (CNB) is a weak CB1 antagonist/CB2 inverse agonist interacting also with GPR55, TRPVR1 vanilloid receptors and μ-opioid receptors [1, 39, 75, 76].

The dose dependent reduction in viability was more pronounced in suspension compared to co-culture. Although not statistically significant, this was reflected by different IC_50_ values under the two culture conditions and the fact that in some co-culture incubations IC_50_ values could not be calculated, in particular where the viability of M2-10B4 cells was not much impacted by the compounds. The protective effect of the microenvironment and of supporting cells in culture, particularly under treatment conditions, is known for CLL and for CLL cells [77–79].

The exceptions observed—RM, CBD, and AM630—can be explained by the toxicity these drugs exerted on the feeder cells. Likewise, healthy PBMC showed IC_50_ values similar to primary leukemic cells alone except for RM which appeared to have much less impact on healthy PBMC compared not only to the mouse fibroblasts but also to neoplastic cells, an observation that has been reported before [33]. In this line, Almestrand reported that normal PBMC subsets did not show significant changes in persons treated with rimonabant for obesity [80] while this drug induced cell death in CB1 expressing primary MCL cells in cell culture [22]. Likewise i*n vitro*, rimonabant, a CB1 antagonist/inverse agonist and μ-opioid antagonist, appeared to influence healthy PBMC to a much lesser degree compared to Jurkat and U937 cells [27]. In the presented study, also AM630, the CB2 antagonist/inverse agonist, had less cytotoxic impact on healthy PBMC compared to primary CLL cells. Together this information suggests that differential or equal cytotoxic impact of cannabinoids on normal and malignant cells may be highly compound specific.

It is noteworthy, that the cytotoxic effect was not associated with either CNR1 and/or CNR2 mRNA levels. Our data differ in this respect from other studies in which the cytotoxic effect of cannabinoids observed in MCL, CLL, and HL cell lines were attributed to the overexpression of cannabinoid receptors in these cells [22, 23, 34] while other, low expressing cell lines were not inhibited [22, 23]. They also suggest that the cytotoxic effects of cannabinoids very likely are mediated by more than one receptor and one mechanism even in cases where molecules are reportedly highly specific. As mentioned above, a certain degree of unspecific action seems to be inherent to the nature of cannabinoids, an aspect that is increasingly reflected by the literature reporting a broad range of cannabinoid activity on a variety of G-protein coupled and other receptors [1, 39–41, 66, 71–74, 81–83].

Cannabinoids supposedly interact with factors in serum which may lead to an inhibition of cellular metabolism, cannabinoid functionality, and reduction in cytotoxicity [66, 84–86]. As described previously [20, 22, 33], we found that particularly low serum concentrations enhanced the reduction in viability under both culture conditions, although less so in co-culture. These serum dependent differences, however, diminished at the highest drug concentrations where the cytotoxic effects of cannabinoids gained impact. While we do not exclude an interaction of cannabinoids with factors in serum causing an attenuation of cannabinoid cytotoxicity, we think that the impact of such effects in the course of CLL therapy is limited. In peripheral blood, under normal serum conditions, higher concentrations of cannabinoids would be required that would also lead to a high degree of toxicity in healthy cells. Under serum reduced conditions, in lymphoid organs, again higher concentrations of cannabinoids would be required since the microenvironment attenuates at least part of the cytotoxic effect of cannabinoids, and, again, healthy cells would also be influenced severely. The net effect under both conditions would be the same: a necessity of higher drug concentrations, a higher portion of healthy cells also being effected, but no real gain in killing CLL cells.

Another feature of cannabinoids should be noted. The ambivalent and variable mechanism of cannabinoid action may cause block or activation of a receptor at low concentrations while high concentrations will lead to cell death [66, 83]. Such bimodal behavior may explain the increases of viability at low concentrations in some experiments.

Interaction with the microenvironment is extremely important for CLL cells and relies to a large part on the CXCR4-CXCL12 axis, which also serves as target for therapies [79, 87]. Based on the blockage of CXCL12 induced chemotaxis and inhibition of similar receptor-ligand pairs in various lineages of peripheral blood cells [17, 88–91], we studied potential synergistic and inhibitory effects of cannabinoids with regard to the standard therapeutic agent fludarabine. Although enhanced reduction in viability could be observed in all experiments, pre-incubation with cannabinoids before addition of fludarabine did not add significantly to the reduction in viability except for the CB1 specific agonist ACEA at the highest concentration. On the other hand, our controls using the CXCR4-specific inhibitor AMD3100 did not lead to a significant synergistic effect, either. This is in contrast to a previous study where pre-treatment with AMD3100 led to a statistically significant reduction of viable CLL cells in co-culture after incubations with different drugs [79]. Although both studies used less than 10 samples for this assay (N = 8 in the Stamatopoulos study, N = 5 in this study), the difference might be attributed to the high biological variability of CLL samples and/or the different type of feeder cells used (mesenchymal stromal cells in the Stamatopoulos study, M2-10B4 mouse fibroblasts in this study) [79].

Although the impact of AMD3100 in co-incubatory experiments was limited, we found this drug to significantly block migration of CLL cells towards CXCL12. In contrast, none of our incubations with cannabinoids led to a significant inhibition of the migratory behavior of primary cells. This indicates that AMD3100 successfully interfered with the CXCR4 receptor in our experiments, but also suggests that the previously reported inhibition of the CXCR4-CXCL12 axis using cannabinoids in different blood cells [17, 89] is not valid in the CLL setting and will most likely not beneficially contribute to therapeutic regimens.

It is still unclear what role the endocannabinoid system my play in cancer and there still is much information to be gathered on how to exploit this system for anti-cancer therapy. At least for MCL some knowledge has accumulated on how cannabinoids effect malignant cells, in addition to the possibility that the endocannabinoid system even may be involved in leukemic development [22, 24, 51, 66, 92]. Also very prominent are the studies evaluating the interference of cannabinoids with cell-cell cross-talk by different chemotaxis pairs [17, 89], which also would constitute an important aspect of targeting therapies.

We, however, could not corroborate these previous findings regarding a substantial inhibition of chemotaxis and a block of the CXCR4-CXCL12 axis by cannabinoids for CLL cells. While we did find that cannabinoids reduced viability of CLL primary cells considerably independent of CNR mRNA expression, we found healthy cells to be affected to the same degree. Thus—and in contrast to other malignancies—our data suggest cannabinoids to be of poor therapeutic potential for treatment of CLL although CNR1 mRNA expression could be determined as novel prognostic marker. Their role in CLL notwithstanding, cannabinoids may still proof useful for anti-tumor therapy in other, selected hematologic malignancies and solid tumors in which the potential of cannabinoids will have to be studied accordingly.

## Supporting Information

S1 Fig No differences in survival of CLL patients in relation to CNR2 mRNA expression. (A) Mean overall survival (OS) for high expressing patients was 196 months vs. 230 months for low expressing patients (N = 107; p = 0.763). (B) Mean treatment free survival (TFS) in CNR2 high and low mRNA expressers was 100 months vs. 135 months in high and low expression groups, respectively (N = 107; p = 0.2290). One hundred and seven patients were included in the analysis, median mRNA expression of CNR2 (3.77) was used as cut-off.
(PDF)

S2 Fig Cytotoxic effect of cannabinoids in combination with fludarabine. CLL primary cells (N = 5) were incubated in triplicates in co-culture with M2-10B4 mouse fibroblasts and incubated for 30 minutes with increasing concentrations of cannabinoids before fludarabine (5 μM) was added. Viability was determined after 48h. Incubations with vehicle served as control. For comparison, cells were incubated with fludarabine alone, with AMD3100 alone, and with AMD3100 in combination with fludarabine (N = 6). Mean values and standard deviations are shown. Hatched lines mark experimental blocks. The synergistic effect of the combination 40 μM ACEA with 5μM fludarabine was significantly different from the effect of 40μM ACEA alone. *p = 0.047. Abbreviation: CNB, (-)-cannabidiol.
(PDF)

S3 Fig Cytotoxic effect of cannabinoids under serum-reduced conditions. PBMC of 5 CLL patients were incubated in triplicates for 48h in increasing compound concentrations at 1%, 2.5%, 5%, and 10% serum containing medium in suspension and in co-culture with M2-10B4 mouse fibroblasts before viability was measured. Mean values and standard deviations are shown. (A) (R)-(+)-methanandamide in suspension and (B) in co-culture. (C) (-)-cannabidiol in suspension and (D) in co-culture. (E) ACEA in suspension and (F) in co-culture. (G) JWH133 in suspension and (H) in co-culture. (I) AM251 in suspension and (J) in co-culture. (K) AM630 in suspension and (L) in co-culture. Note different scales on x- and y-axes.
(PDF)

S4 Fig Cytotoxicity of cannabinoids in relation to CNR1 mRNA expression. PBMC from CLL patients were incubated in triplicates in increasing compound concentrations in suspension and co-culture with M2-10B4 mouse fibroblast cells for 48h before viability was measured. (A) (R)-(+)-methanandamide (N = 10). (B) (-)-cannabidiol (N = 18). (C) ACEA (N = 16). (D) JWH133 (N = 16). (E) AM251 (N = 16). (F) AM630 (N = 16). The x-axis shows the measured mRNA expression for each CLL sample tested (healthy CD19 sorted cells set as 1) from highest (left) to lowest (right) expression. Absent values may indicate that i) sample was not tested, or ii) IC_50_ could not be calculated, or iii) 50% viability reduction could not be achieved. Note different scales on Y-axis for A and D.
(PDF)

S5 Fig Cytotoxicity of cannabinoids in relation to CNR2 mRNA expression. PBMC from CLL patients were incubated in triplicates in increasing compound concentrations in suspension and co-culture with M2-10B4 mouse fibroblast cells for 48h before viability was measured. (A) (R)-(+)-methanandamide (N = 10). (B) (-)-cannabidiol (N = 18). (C) ACEA (N = 16). (D) JWH133 (N = 16). (E) AM251 (N = 16). (F) AM630 (N = 16). The x-axis shows the measured mRNA expression for each CLL sample tested (healthy CD19 sorted cells set as 1) from highest (left) to lowest (right) expression. Absent values may indicate that i) sample was not tested, or ii) IC_50_ could not be calculated, or iii) 50% viability reduction could not be achieved. Note different scales on Y-axis for A and D.
(PDF)

S6 Fig Impact of cannabinoids on CLL cell migration in unsorted PBMC. Primary cells of 5 CLL patients were pre-incubated with cannabinoids before being transferred to transwell plates and incubated for 4h for migration. Control experiments included CXCL12 alone (control), no CXCL12 (control w/o CXCL12), incubation with vehicle (DMSO, ethanol), and incubation with the CXCR4 inhibitor AMD3100. CLL cells were incubated either with agonist (ACEA, JWH133) or antagonist (AM251, AM630) before migration. In addition, cells were treated with antagonist before agonist incubation before migration was allowed (CB1: AMS251&ACEA; CB2: AM630&JWH133). Bars represent mean values of migration indices + standard deviations, hatched lines indicate experimental blocks.* p = 0.0016; ** p<0.0001.
(PDF)

S1 Table Clinical characteristics of the patients used in cannabinoid receptor mRNA analysis. (PDF)

S2 Table Compound concentrations and duration of incubations before initiation of migration experiments. (PDF)

S3 Table Comparison of patient characteristics between CNR2 high and low mRNA expressing groups. (PDF)

## Abbreviations

CLL: chronic lymphocytic leukemia
CNB: (-)-cannabidiol
CNR1/2, CB1/2: cannabinoid receptor 1/2
HL: Hodgkin lymphoma
MCL: mantle cell lymphoma
PBMC: peripheral blood mononuclear cells
RM: R-(+)-methanandamide

## References

[1] Pertwee RG, Howlett AC, Abood ME, Alexander SP, Di Marzo V, Elphick MR, et al International Union of Basic and Clinical Pharmacology. LXXIX. Cannabinoid receptors and their ligands: beyond CB1 and CB2. Pharmacol Rev. 2010; 62(4):588–631. 10.1124/pr.110.003004 21079038 PMC2993256

[2] Guzmán M. Cannabinoids: potential anticancer agents. Nat Rev Cancer. 2003; 3(10):745–755. 14570037 10.1038/nrc1188

[3] Atwood BK, Mackie K. CB2: a cannabinoid receptor with an identity crisis. Br J Pharmacol. 2010; 160(3):467–479. 10.1111/j.1476-5381.2010.00729.x 20590558 PMC2931549

[4] Velasco G, Sanchez C, Guzman M. Towards the use of cannabinoids as antitumour agents. Nat Rev Cancer. 2012; 12:436–444. 10.1038/nrc3247 22555283

[5] Engel MA, Kellermann CA, Burnat G, Hahn EG, Rau T, Konturek PC. Mice lacking cannabinoid CB1-, CB2-receptors or both receptors show increased susceptibility to trinitrobenzene sulfonic acid (TNBS)-induced colitis. J Physiol Pharmacol. 2010; 61(1):89–97. 20228420

[6] Ortega-Alvaro A, Aracil-Fernández A, García-Gutiérrez MS, Navarrete F, Manzanares J. Deletion of CB2 cannabinoid receptor induces schizophrenia-related behaviors in mice. Neuropsychopharmacology. 2011; 36(7):1489–1504. 10.1038/npp.2011.34 21430651 PMC3096817

[7] Duncan M, Galic MA, Wang A, Chambers AP, McCafferty DM, McKay DM, et al Cannabinoid 1 receptors are critical for the innate immune response to TLR4 stimulation. Am J Physiol Regul Integr Comp Physiol. 2013; 305(3):R224–R231. 10.1152/ajpregu.00104.2013 23739343

[8] Rodriguez-Arias M, Navarrete F, Daza-Losada M, Navarro D, Aguilar MA, Berbel P, et al CB1 cannabinoid receptor-mediated aggressive behavior. Neuropharmacology. 2013; 75:172–180. 10.1016/j.neuropharm.2013.07.013 23916480

[9] Suárez-Pinilla P, López-Gil J, Crespo-Facorro B. Immune system: a possible nexus between cannabinoids and psychosis. Brain Behav Immun. 2014; 40:269–282. 10.1016/j.bbi.2014.01.018 24509089

[10] Xie H, Sun X, Piao Y, Jegga AG, Handwerger S, Ko MS, et al Silencing or amplification of endocannabinoid signaling in blastocysts via CB1 compromises trophoblast cell migration. J Biol Chem. 2012; 287(38):32288–32297. 10.1074/jbc.M112.381145 22833670 PMC3442559

[11] Liu YJ, Fan HB, Jin Y, Ren CG, Jia XE, Wang L, et al Cannabinoid receptor 2 suppresses leukocyte inflammatory migration by modulating the JNK/c-Jun/Alox5 pathway. J Biol Chem. 2013; 288(19):13551–13562. 10.1074/jbc.M113.453811 23539630 PMC3650391

[12] Saez TM, Aronne MP, Caltana L, Brusco AH. Prenatal exposure to the CB1 and CB2 cannabinoid receptor agonist WIN 55,212–2 alters migration of early-born glutamatergic neurons and GABAergic interneurons in the rat cerebral cortex. J Neurochem. 2014; 129(4):637–648. 10.1111/jnc.12636 24329778

[13] Jordà MA, Verbakel SE, Valk PJ, Vankan-Berkhoudt YV, Maccarrone M, Finazzi-Agrò A, et al Hematopoietic cells expressing the peripheral cannabinoid receptor migrate in response to the endocannabinoid 2-arachidonoylglycerol. Blood. 2002; 99(8):2786–2793. 11929767 10.1182/blood.v99.8.2786

[14] Pereira JP, An J, Xu Y, Huang Y, Cyster JG. Cannabinoid receptor 2 mediates the retention of immature B cells in bone marrow sinusoids. Nat Immunol. 2009; 10(4):403–411. 10.1038/ni.1710 19252491 PMC2768754

[15] Basu S, Ray A, Dittel BN. Cannabinoid receptor 2 is critical for the homing and retention of marginal zone B lineage cells and for efficient T-independent immune responses. J Immunol. 2011; 187(11):5720–5732. 10.4049/jimmunol.1102195 22048769 PMC3226756

[16] Muppidi JR, Arnon TI, Bronevetsky Y, Veerapen N, Tanaka M, Besra GS, et al Cannabinoid receptor 2 positions and retains marginal zone B cells within the splenic marginal zone. J Exp Med. 2011; 208(10):1941–1948. 10.1084/jem.20111083 21875957 PMC3182059

[17] Tanikawa T, Kurohane K, Imai Y. Regulatory effect of cannabinoid receptor agonist on chemokine-induced lymphocyte chemotaxis. Biol Pharm Bull. 2011; 34(7):1090–1093. 21720018 10.1248/bpb.34.1090

[18] Möhle R, Bautz F, Denzlinger C, Kanz L. Transendothelial migration of hematopoietic progenitor cells. Role of chemotactic factors. Ann N Y Acad Sci. 2001; 938:26–35. 11458515 10.1111/j.1749-6632.2001.tb03571.x

[19] Patinkin D, Milman G, Breuer A, Fride E, Mechoulam R. Endocannabinoids as positive or negative factors in hematopoietic cell migration and differentiation. Eur J Pharmacol. 2008; 595(1–3):1–6. 10.1016/j.ejphar.2008.05.002 18778813

[20] McKallip RJ, Lombard C, Fisher M, Martin BR, Ryu S, Grant S, et al Targeting CB2 cannabinoid receptors as a novel therapy to treat malignant lymphoblastic disease. Blood. 2002; 100(2):627–634. 12091357 10.1182/blood-2002-01-0098

[21] Massi P, Vaccani A, Ceruti S, Colombo A, Abbracchio MP, Parolaro D. Antitumor effects of cannabidiol, a nonpsychoactive cannabinoid, on human glioma cell lines. J Pharmacol Exp Ther. 2004; 308(3):838–845. 14617682 10.1124/jpet.103.061002

[22] Flygare J, Gustafsson K, Kimby E, Christensson B, Sander B. Cannabinoid receptor ligands mediate growth inhibition and cell death in mantle cell lymphoma. FEBS Lett. 2005; 579(30):6885–6889. 16337199 10.1016/j.febslet.2005.11.020

[23] Gustafsson K, Wang X, Severa D, Eriksson M, Kimby E, Merup M, et al Expression of cannabinoid receptors type 1 and type 2 in non-Hodgkin lymphoma: growth inhibition by receptor activation. Int J Cancer. 2008; 123(5):1025–1033. 10.1002/ijc.23584 18546271

[24] Gustafsson SB, Lindgren T, Jonsson M, Jacobsson SO. Cannabinoid receptor-independent cytotoxic effects of cannabinoids in human colorectal carcinoma cells: synergism with 5-fluorouracil. Cancer Chemother Pharmacol. 2009; 63(4):691–701. 10.1007/s00280-008-0788-5 18629502

[25] Olea-Herrero N, Vara D, Malagarie-Cazenave S, Díaz-Laviada I. Inhibition of human tumour prostate PC-3 cell growth by cannabinoids R(+)-Methanandamide and JWH-015: involvement of CB2. Br J Cancer. 2009; 101(6):940–950. 10.1038/sj.bjc.6605248 19690545 PMC2743360

[26] Cudaback E, Marrs W, Moeller T, Stella N. The expression level of CB1 and CB2 receptors determines their efficacy at inducing apoptosis in astrocytomas. PLoS ONE. 2010; 5(1):e8702-. 10.1371/journal.pone.0008702 20090845 PMC2806825

[27] Gallotta D, Nigro P, Cotugno R, Gazzerro P, Bifulco M, Belisario MA. Rimonabant-induced apoptosis in leukemia cell lines: activation of caspase-dependent and -independent pathways. Biochem Pharmacol. 2010; 80:370–380. 10.1016/j.bcp.2010.04.023 20417624

[28] Marcu JP, Christian RT, Lau D, Zielinski AJ, Horowitz MP, Lee J, et al Cannabidiol enhances the inhibitory effects of delta9-tetrahydrocannabinol on human glioblastoma cell proliferation and survival. Mol Cancer Ther. 2010; 9(1):180–189. 10.1158/1535-7163.MCT-09-0407 20053780 PMC2806496

[29] Nasser MW, Qamri Z, Deol YS, Smith D, Shilo K, Zou X, et al Crosstalk between chemokine receptor CXCR4 and cannabinoid receptor CB2 in modulating breast cancer growth and invasion. PLoS One. 2011; 6(9):e23901-. 10.1371/journal.pone.0023901 21915267 PMC3168464

[30] Preet A, Qamri Z, Nasser MW, Prasad A, Shilo K, Zou X, et al Cannabinoid receptors, CB1 and CB2, as novel targets for inhibition of non-small cell lung cancer growth and metastasis. Cancer Prev Res (Phila). 2011; 4(1):65–75. 21097714 10.1158/1940-6207.CAPR-10-0181 PMC3025486

[31] Shrivastava A, Kuzontkoski PM, Groopman JE, Prasad A. Cannabidiol induces programmed cell death in breast cancer cells by coordinating the cross-talk between apoptosis and autophagy. Mol Cancer Ther. 2011; 10(7):1161–1172. 10.1158/1535-7163.MCT-10-1100 21566064

[32] Martínez-Martínez E, Gómez I, Martín P, Sánchez A, Román L, Tejerina E, et al Cannabinoids receptor type 2, CB2, expression correlates with human colon cancer progression and predicts patient survival. Oncoscience. 2015; 2(2):131–141. 25859556 10.18632/oncoscience.119 PMC4381706

[33] Gustafsson K, Christensson B, Sander B, Flygare J. Cannabinoid receptor-mediated apoptosis induced by R(+)-methanandamide and Win55,212–2 is associated with ceramide accumulation and p38 activation in mantle cell lymphoma. Mol Pharmacol. 2006; 70(5):1612–1620. 16936228 10.1124/mol.106.025981

[34] Benz AH, Renné C, Maronde E, Koch M, Grabiec U, Kallendrusch S, et al Expression and functional relevance of cannabinoid receptor 1 in Hodgkin lymphoma. PLoS ONE. 2013; 8(12):e81675-. 10.1371/journal.pone.0081675 24349109 PMC3857220

[35] Guida M, Ligresti A, De Filippis D, D'Amico A, Petrosino S, Cipriano M, et al The levels of the endocannabinoid receptor CB2 and its ligand 2-arachidonoylglycerol are elevated in endometrial carcinoma. Endocrinology. 2010; 151(3):921–928. 10.1210/en.2009-0883 20133454

[36] Farsandaj N, Ghahremani MH, Ostad SN. Role of cannabinoid and vanilloid receptors in invasion of human breast carcinoma cells. J Environ Pathol Toxicol Oncol. 2012; 31(4):377–387. 23394450 10.1615/jenvironpatholtoxicoloncol.2013005859

[37] Pourkhalili N, Ghahremani MH, Farsandaj N, Tavajohi S, Majdzadeh M, Parsa M, et al Evaluation of anti-invasion effect of cannabinoids on human hepatocarcinoma cells. Toxicology Mechanisms and Methods. 2013; 23(2):120–126. 10.3109/15376516.2012.730559 22978792

[38] Ligresti A, Moriello AS, Starowicz K, Matias I, Pisanti S, De Petrocellis L, et al Antitumor activity of plant cannabinoids with emphasis on the effect of cannabidiol on human breast carcinoma. J Pharmacol Exp Ther. 2006; 318(3):1375–1387. 16728591 10.1124/jpet.106.105247

[39] Kathmann M, Flau K, Redmer A, Tränkle C, Schlicker E. Cannabidiol is an allosteric modulator at mu- and delta-opioid receptors. Naunyn Schmiedebergs Arch Pharmacol. 2006; 372(5):354–361. 16489449 10.1007/s00210-006-0033-x

[40] Seely KA, Brents LK, Franks LN, Rajasekaran M, Zimmerman SM, Fantegrossi WE, et al AM-251 and rimonabant act as direct antagonists at mu-opioid receptors: implications for opioid/cannabinoid interaction studies. Neuropharmacology. 2012; 63(5):905–915. 10.1016/j.neuropharm.2012.06.046 22771770 PMC3408547

[41] Ryberg E, Larsson N, Sjögren S, Hjorth S, Hermansson NO, Leonova J, et al The orphan receptor GPR55 is a novel cannabinoid receptor. Br J Pharmacol. 2007; 152(7):1092–1101. 17876302 10.1038/sj.bjp.0707460 PMC2095107

[42] Xu X, Liu Y, Huang S, Liu G, Xie C, Zhou J, et al Overexpression of cannabinoid receptors CB1 and CB2 correlates with improved prognosis of patients with hepatocellular carcinoma. Cancer Genet Cytogenet. 2006; 171(1):31–38. 17074588 10.1016/j.cancergencyto.2006.06.014

[43] Theocharis S, Giaginis C, Alexandrou P, Rodriguez J, Tasoulas J, Danas E, et al Evaluation of cannabinoid CB1 and CB2 receptors expression in mobile tongue squamous cell carcinoma: associations with clinicopathological parameters and patients' survival. Tumour Biol. 2015;:, in press. 10.1007/s13277-015-4182-8 26459312

[44] Jung CK, Kang WK, Park JM, Ahn HJ, Kim SW, Taek Oh S, et al Expression of the cannabinoid type I receptor and prognosis following surgery in colorectal cancer. Oncology Letters. 2013; 5(3):870–876. 23426698 10.3892/ol.2012.1081 PMC3576207

[45] Fowler CJ, Hammarsten P, Bergh A. Tumour Cannabinoid CB(1) receptor and phosphorylated epidermal growth factor receptor expression are additive prognostic markers for prostate cancer. PLoS ONE. 2010; 5(12):e15205-. 10.1371/journal.pone.0015205 21203460 PMC3009725

[46] Gustafsson SB, Palmqvist R, Henriksson ML, Dahlin AM, Edin S, Jacobsson SO, et al High tumour cannabinoid CB1 receptor immunoreactivity negatively impacts disease-specific survival in stage II microsatellite stable colorectal cancer. PLoS ONE. 2011; 6(8):e23003-. 10.1371/journal.pone.0023003 21901119 PMC3161987

[47] Pérez-Gómez E, Andradas C, Blasco-Benito S, Caffarel MM, García-Taboada E, Villa-Morales M, et al Role of cannabinoid receptor CB2 in HER2 pro-oncogenic signaling in breast cancer. J Natl Cancer Inst. 2015; 107(6):djv077-. 10.1093/jnci/djv077 25855725

[48] Klein Nulent TJ, Van Diest PJ, van der Groep P, Leusink FK, Kruitwagen CL, Koole R, et al Cannabinoid receptor-2 immunoreactivity is associated with survival in squamous cell carcinoma of the head and neck. British Journal of Oral and Maxillofacial Surgery. 2013; 51(7):604–609. 10.1016/j.bjoms.2013.03.015 23601830

[49] Piszcz JA, Lemancewicz D, Kloczko J, Dzieciol J, Rusak M, Dabrowska M. Cannabinoid receptors expression in bone marrow trephine biopsy of chronic lymphocytic leukaemia patients treated with purine analogues. Exp Oncol. 2007; 29(3):221–225. 18004250

[50] Rayman N, Lam KH, van der Holt B, Koss C, van Leeuwen J, Budel LM, et al The expression of the peripheral cannabinoid receptor CB2 has no effect on clinical outcome in diffuse large B-cell lymphomas. Eur J Haematol. 2011; 86(6):466–476. 10.1111/j.1600-0609.2011.01596.x 21457344

[51] Wasik AM, Nygren L, Almestrand S, Zong F, Flygare J, Wennerholm SB, et al Perturbations of the endocannabinoid system in mantle cell lymphoma: correlations to clinical and pathological features. Oncoscience. 2014; 1(8):550–557. 25594062 10.18632/oncoscience.77 PMC4278325

[52] Balakrishnan K, Gandhi V. Bcl-2 antagonists: a proof of concept for CLL therapy. Invest New Drugs. 2013; 31(5):1384–1394. 10.1007/s10637-013-0002-4 23907405 PMC4152228

[53] Hoellenriegel J, Zboralski D, Maasch C, Rosin NY, Wierda WG, Keating MJ, et al The Spiegelmer NOX-A12, a novel CXCL12 inhibitor, interferes with chronic lymphocytic leukemia cell motility and causes chemosensitization. Blood. 2014; 123(7):1032–1039. 10.1182/blood-2013-03-493924 24277076 PMC4123413

[54] Jones JA, Byrd JC. How will B-cell-receptor-targeted therapies change future CLL therapy? Blood. 2014; 123(10):1455–1460. 10.1182/blood-2013-09-453092 24394667 PMC3945859

[55] Woyach JA, Furman RR, Liu TM, Ozer HG, Zapatka M, Ruppert AS, et al Resistance mechanisms for the Bruton's tyrosine kinase inhibitor ibrutinib. N Engl J Med. 2014; 370(24):2286–2294. 10.1056/NEJMoa1400029 24869598 PMC4144824

[56] Cao Y, Hunter ZR, Liu X, Xu L, Yang G, Chen J, et al The WHIM-like CXCR4(S338X) somatic mutation activates AKT and ERK, and promotes resistance to ibrutinib and other agents used in the treatment of Waldenstrom's Macroglobulinemia. Leukemia. 2015; 29:169–176. 10.1038/leu.2014.187 24912431

[57] Schuh A, Becq J, Humphray S, Alexa A, Burns A, Clifford R, et al Monitoring chronic lymphocytic leukemia progression by whole genome sequencing reveals heterogeneous clonal evolution patterns. Blood. 2012; 120(20):4191–4196. 10.1182/blood-2012-05-433540 22915640

[58] Landau DA, Carter SL, Stojanov P, McKenna A, Stevenson K, Lawrence MS, et al Evolution and impact of subclonal mutations in chronic lymphocytic leukemia. Cell. 2013; 152(4):714–726. 10.1016/j.cell.2013.01.019 23415222 PMC3575604

[59] Puente XS, López-Otín C. The evolutionary biography of chronic lymphocytic leukemia. Nat Genet. 2013; 45(3):229–231. 10.1038/ng.2556 23435090

[60] Livak KJ, Schmittgen TD. Analysis of relative gene expression data using real-time quantitative PCR and the 2(-Delta Delta C(T)) Method. Methods. 2001; 25(4):402–408. 11846609 10.1006/meth.2001.1262

[61] Song ZH, Zhong M. CB1 cannabinoid receptor-mediated cell migration. J Pharmacol Exp Ther. 2000; 294(1):204–209. 10871313

[62] He F, Qiao ZH, Cai J, Pierce W, He DC, Song ZH. Involvement of the 90-kDa heat shock protein (Hsp-90) in CB2 cannabinoid receptor-mediated cell migration: a new role of Hsp-90 in migration signaling of a G protein-coupled receptor. Mol Pharmacol. 2007; 72(5):1289–1300. 17698952 10.1124/mol.107.036566

[63] Gentilini D, Besana A, Vigano P, Dalino P, Vignali M, Melandri M, et al Endocannabinoid system regulates migration of endometrial stromal cells via cannabinoid receptor 1 through the activation of PI3K and ERK1/2 pathways. Fertil Steril. 2010; 93(8):2588–2593. 10.1016/j.fertnstert.2010.02.006 20303477

[64] Matsuda LA, Lolait SJ, Brownstein MJ, Young AC, Bonner TI. Structure of a cannabinoid receptor and functional expression of the cloned cDNA. Nature. 1990; 346(6284):561–564. 2165569 10.1038/346561a0

[65] Munro S, Thomas KL, Abu-Shaar M. Molecular characterization of a peripheral receptor for cannabinoids. Nature. 1993; 365(6441):61–65. 7689702 10.1038/365061a0

[66] Wasik AM, Christensson B, Sander B. The role of cannabinoid receptors and the endocannabinoid system in mantle cell lymphoma and other non-Hodgkin lymphomas. Semin Cancer Biol. 2011; 21:313–321. 10.1016/j.semcancer.2011.10.004 22024769

[67] Rayman N, Lam KH, Van Leeuwen J, Mulder AH, Budel LM, Löwenberg B, et al The expression of the peripheral cannabinoid receptor on cells of the immune system and non-Hodgkin's lymphomas. Leuk Lymphoma. 2007; 48(7):1389–1399. 17613768 10.1080/10428190701377030

[68] Michalski CW, Oti FE, Erkan M, Sauliunaite D, Bergmann F, Pacher P, et al Cannabinoids in pancreatic cancer: correlation with survival and pain. Int J Cancer. 2008; 122(4):742–750. 17943729 10.1002/ijc.23114 PMC2225529

[69] Grimsey NL, Goodfellow CE, Scotter EL, Dowie MJ, Glass M, Graham ES. Specific detection of CB1 receptors; cannabinoid CB1 receptor antibodies are not all created equal!. J Neurosci Methods. 2008; 171(1):78–86. 10.1016/j.jneumeth.2008.02.014 18406468

[70] Graham ES, Angel CE, Schwarcz LE, Dunbar PR, Glass M. Detailed characterisation of CB2 receptor protein expression in peripheral blood immune cells from healthy human volunteers using flow cytometry. Int J Immunopathol Pharmacol. 2010; 23(1):25–34. 20377992 10.1177/039463201002300103

[71] Patil M, Patwardhan A, Salas MM, Hargreaves KM, Akopian AN. Cannabinoid receptor antagonists AM251 and AM630 activate TRPA1 in sensory neurons. Neuropharmacology. 2011; 61(4):778–788. 10.1016/j.neuropharm.2011.05.024 21645531 PMC3130079

[72] Kapur A, Zhao P, Sharir H, Bai Y, Caron MG, Barak LS, et al Atypical responsiveness of the orphan receptor GPR55 to cannabinoid ligands. J Biol Chem. 2009; 284:29817–29827. 10.1074/jbc.M109.050187 19723626 PMC2785612

[73] McDougall JJ, Yu V, Thomson J. *In vivo* effects of CB_2_ receptor-selective cannabinoids on the vasculature of normal and arthritic rat knee joints. Br J Pharmacol. 2008; 153:358–366. 17982474 10.1038/sj.bjp.0707565 PMC2219539

[74] Taylor L, Christou I, Kapellos TS, Buchan A, Brodermann MH, Gianella-Borradori M, et al Primary macrophage chemotaxis induced by cannabinoid receptor 2 agonists occurs independently of the CB_2_ receptor. Scientific Reports. 2015; 5:10682-. 10.1038/srep10682 26033291 PMC4451551

[75] Costa B, Giagnoni G, Franke C, Trovato AE, Colleoni M. Vanilloid TRPV1 receptor mediates the antihyperalgesic effect of the nonpsychoactive cannabinoid, cannabidiol, in a rat model of acute inflammation. Br J Pharmacol. 2004; 143(2):247–250. 15313881 10.1038/sj.bjp.0705920 PMC1575333

[76] Thomas A, Baillie GL, Phillips AM, Razdan RK, Ross RA, Pertwee RG. Cannabidiol displays unexpectedly high potency as an antagonist of CB1 and CB2 receptor agonists in vitro. Br J Pharmacol. 2007; 150(5):613–623. 17245363 10.1038/sj.bjp.0707133 PMC2189767

[77] Kurtova AV, Balakrishnan K, Chen R, Ding W, Schnabl S, Quiroga MP, et al Diverse marrow stromal cells protect CLL cells from spontaneous and drug-induced apoptosis: development of a reliable and reproducible system to assess stromal cell adhesion-mediated drug resistance. Blood. 2009; 114:4441–4450. 10.1182/blood-2009-07-233718 19762485 PMC4081374

[78] Quiroga MP, Balakrishnan K, Kurtova AV, Sivina M, Keating MJ, Wierda WG, et al B cell antigen receptor signaling enhances chronic lymphocytic leukemia cell migration and survival: specific targeting with a novel Syk inhibitor, R406. Blood. 2009; 114:1029–1037. 19491390 10.1182/blood-2009-03-212837 PMC4916941

[79] Stamatopoulos B, Meuleman N, De Bruyn C, Pieters K, Mineur P, Le Roy C, et al AMD3100 disrupts the cross-talk between chronic lymphocytic leukemia cells and a mesenchymal stromal or nurse-like cell -based microenvironment: preclinical evidence for its association with chronic lymphocytic leukemia treatments. Haematologica. 2012; 97:608–615. 10.3324/haematol.2011.052779 22058221 PMC3347654

[80] Almestrand S, Wang X, Jeppsson-Ahlberg A, Nordgren M, Flygare J, Christensson B, et al Influence of rimonabant treatment on peripheral blood mononuclear cells; flow cytometry analysis and gene expression profiling. PeerJ. 2015; 3:e1056-. 10.7717/peerj.1056 26157624 PMC4493638

[81] Powles T, te Poele R, Shamash J, Chaplin T, Propper D, Joel S, et al Cannabis-induced cytotoxicity in leukemic cell lines: the role of the cannabinoid receptors and the MAPK pathway. Blood. 2005; 105(3):1214–1221. 15454482 10.1182/blood-2004-03-1182

[82] Fogli S, Nieri P, Chicca A, Adinolfi B, Mariotti V, Iacopetti P, et al Cannabinoid derivatives induce cell death in pancreatic MIA PaCa-2 cells via a receptor-independent mechanism. FEBS Lett. 2006; 580(7):1733–1739. 16500647 10.1016/j.febslet.2006.02.024

[83] Ramer R, Hinz B. New insights into antimetastatic and antiangiogenic effects of cannabinoids. International Review of Cell and Molecular Biology. 2015; 314:43–116. 10.1016/bs.ircmb.2014.10.005 25619715

[84] Tang JL, Lancz G, Specter S. Delta-9-tetrahydrocannabinol-(THC)-mediated inhibition of macrophage macromolecular metabolism is antagonized by human serum proteins and by cell surface proteins. International Journal of Immunopharmacology. 1993; 15(6):665–672. 8407051 10.1016/0192-0561(93)90139-p

[85] Bojesen IN, Hansen HS. Binding of anandamide to bovine serum albumin. J Lipid Res. 2003; 44(9):1790–1794. 12837852 10.1194/jlr.M300170-JLR200

[86] Richardson SJ, Widmer M, Zajicek J, Rule SA. Physiological doses of cannabinoids do not adversely affect MCL viability. Leuk Lymphoma. 2007; 48(9):1855–1857. 17786725 10.1080/10428190701534465

[87] Burger JA. Chemokines and chemokine receptors in chronic lymphocytic leukemia (CLL): from understanding the basics towards therapeutic targeting. Semin Cancer Biol. 2010; 20(6):424–430. 20883788 10.1016/j.semcancer.2010.09.005

[88] Kishimoto S, Muramatsu M, Gokoh M, Oka S, Waku K, Sugiura T. Endogenous cannabinoid receptor ligand induces the migration of human natural killer cells. J Biochem (Tokyo). 2005; 137(2):217–223. 15749836 10.1093/jb/mvi021

[89] Ghosh S, Preet A, Groopman JE, Ganju RK. Cannabinoid receptor CB2 modulates the CXCL12/CXCR4-mediated chemotaxis of T lymphocytes. Mol Immunol. 2006; 43(14):2169–2179. 16503355 10.1016/j.molimm.2006.01.005

[90] Kishimoto S, Oka S, Gokoh M, Sugiura T. Chemotaxis of human peripheral blood eosinophils to 2-arachidonoylglycerol: comparison with other eosinophil chemoattractants. Int Arch Allergy Immunol. 2006; 140 Suppl 1:3–7. 16772720 10.1159/000092704

[91] Montecucco F, Burger F, Mach F, Steffens S. CB2 cannabinoid receptor agonist JWH-015 modulates human monocyte migration through defined intracellular signaling pathways. Am J Physiol Heart Circ Physiol. 2008; 294(3):H1145–H1155. 10.1152/ajpheart.01328.2007 18178718

[92] Wasik AM, Sander B. Cannabinoid receptors in mantle cell lymphoma. Cell Cycle. 2015; 14(3):291–292. 10.1080/15384101.2015.1006536 25590415 PMC4353235

