# Supplementary figures and images for: Cannabinoid Receptors Are Overexpressed in CLL but of Limited Potential for Therapeutic Exploitation
Source: PLoS One. 2016 Jun 1;11(6):e0156693. doi: 10.1371/journal.pone.0156693 (PMC4889125; doi:10.1371/journal.pone.0156693)

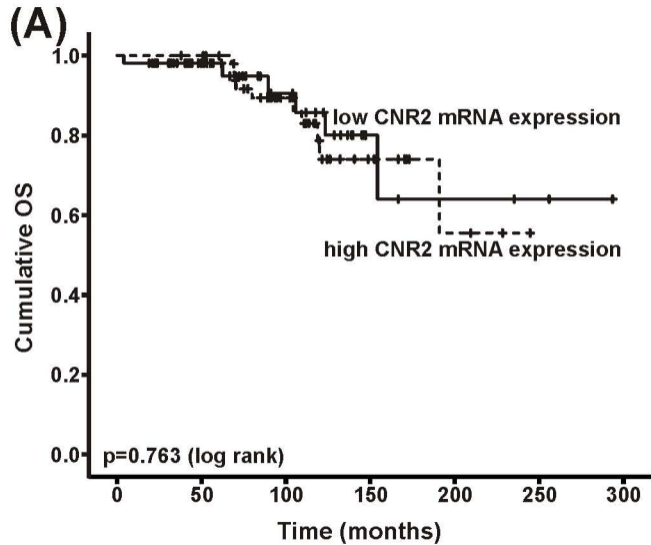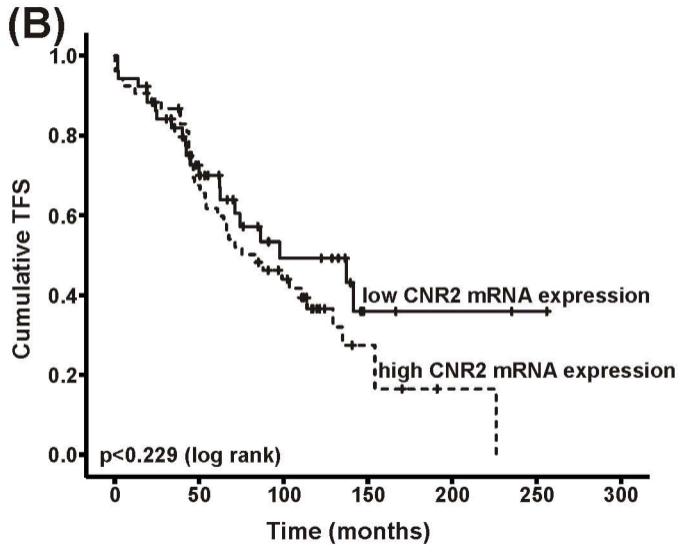

Supplement: S1 Fig — (A) Mean overall survival (OS) for high expressing patients was 196 months vs. 230 months for low expressing patients (N = 107; p = 0.763). (B) Mean treatment free survival (TFS) in CNR2 high and low mRNA expressers was 100 months vs. 135 months in high and low expression groups, respectively (N = 107; p = 0.2290). One hundred and seven patients were included in the analysis, median mRNA expression of CNR2 (3.77) was used as cut-off. (PDF) [file pone.0156693.s001.pdf]

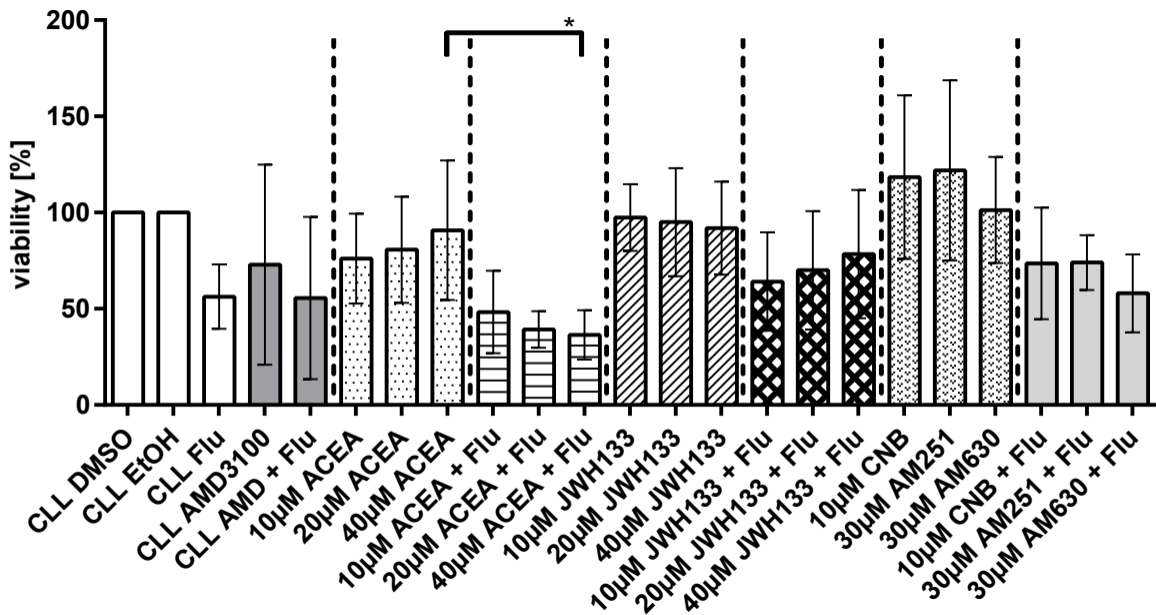

Supplement: S2 Fig — CLL primary cells (N = 5) were incubated in triplicates in co-culture with M2-10B4 mouse fibroblasts and incubated for 30 minutes with increasing concentrations of cannabinoids before fludarabine (5 μM) was added. Viability was determined after 48h. Incubations with vehicle served as control. For comparison, cells were incubated with fludarabine alone, with AMD3100 alone, and with AMD3100 in combination with fludarabine (N = 6). Mean values and standard deviations are shown. Hatched lines mark experimental blocks. The synergistic effect of the combination 40 μM ACEA with 5μM fludarabine was significantly different from the effect of 40μM ACEA alone. *p = 0.047. Abbreviation: CNB, (-)-cannabidiol. (PDF) [file pone.0156693.s002.pdf]

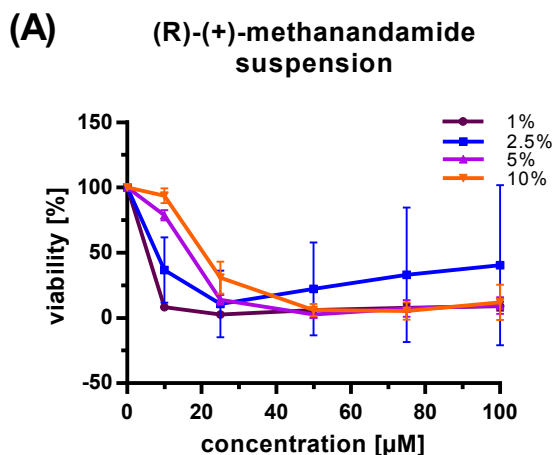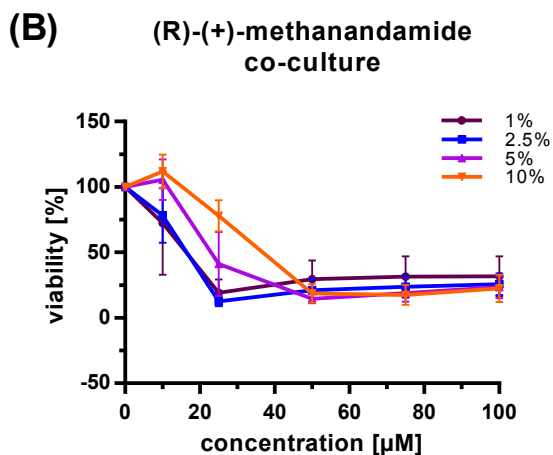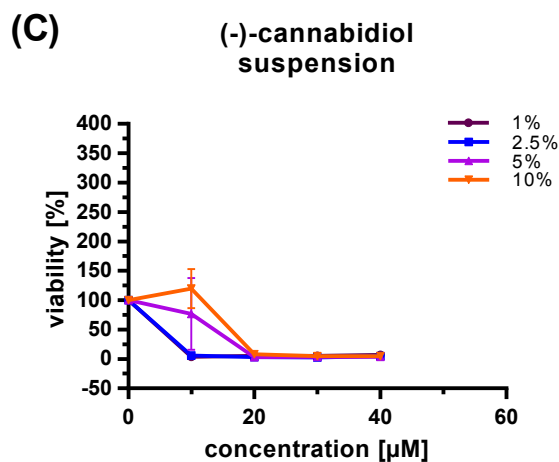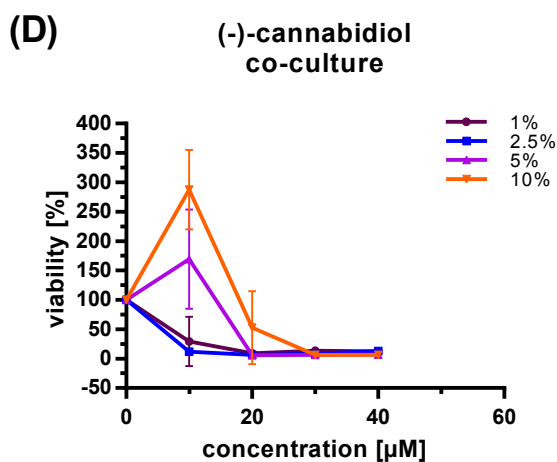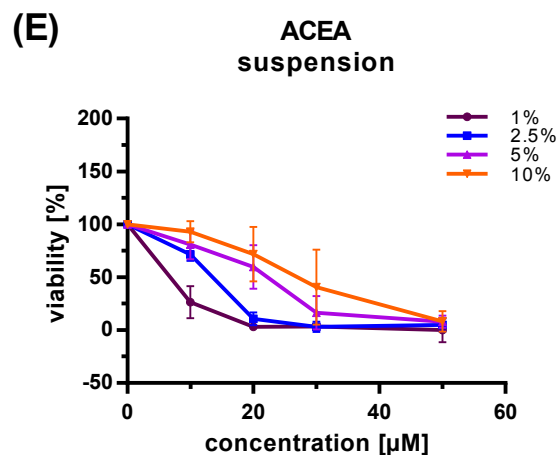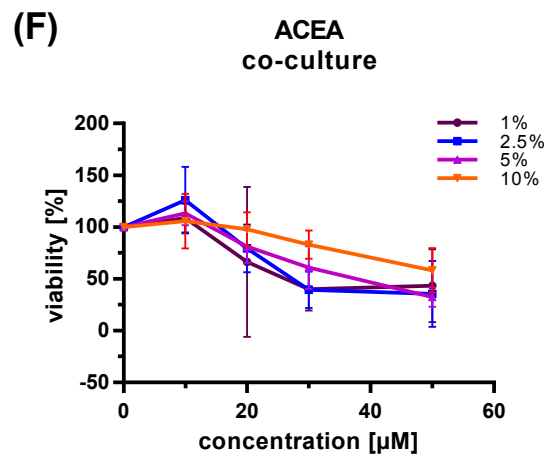

**(G)****JWH133  
suspension**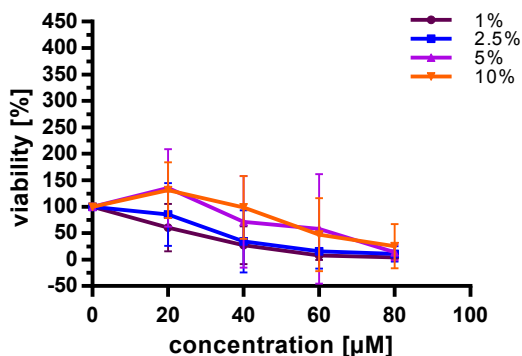**(H)****JWH133  
co-culture**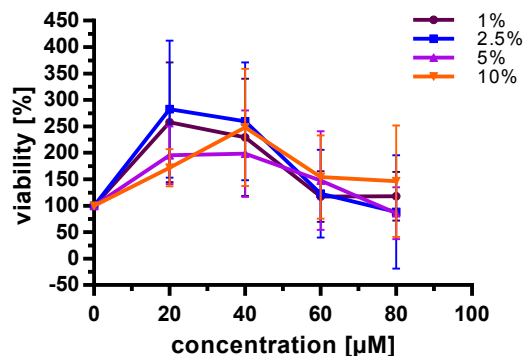**(I)****AM251  
suspension**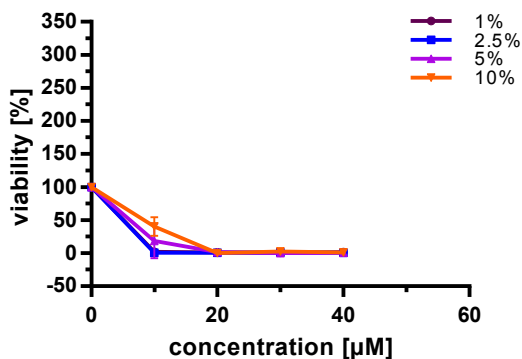**(J)****AM251  
co-culture**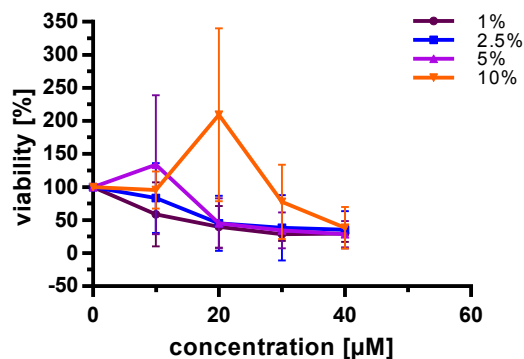**(K)****AM630  
suspension**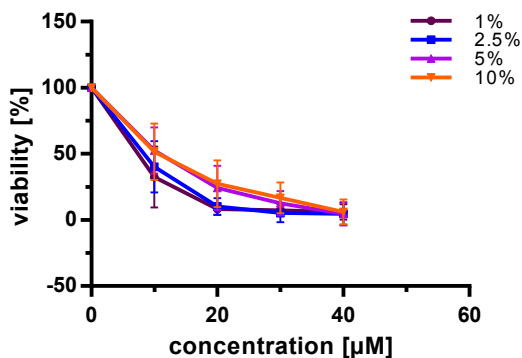**(L)****AM630  
co-culture**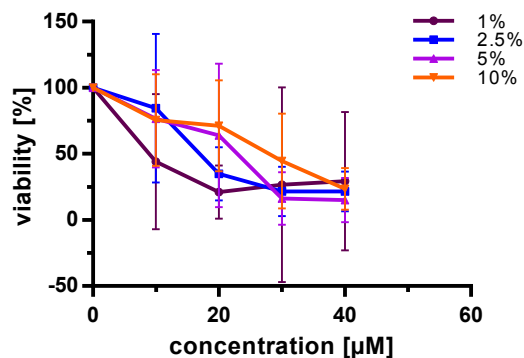

Supplement: S3 Fig — PBMC of 5 CLL patients were incubated in triplicates for 48h in increasing compound concentrations at 1%, 2.5%, 5%, and 10% serum containing medium in suspension and in co-culture with M2-10B4 mouse fibroblasts before viability was measured. Mean values and standard deviations are shown. (A) (R)-(+)-methanandamide in suspension and (B) in co-culture. (C) (-)-cannabidiol in suspension and (D) in co-culture. (E) ACEA in suspension and (F) in co-culture. (G) JWH133 in suspension and (H) in co-culture. (I) AM251 in suspension and (J) in co-culture. (K) AM630 in suspension and (L) in co-culture. Note different scales on x- and y-axes. (PDF) [file pone.0156693.s003.pdf]

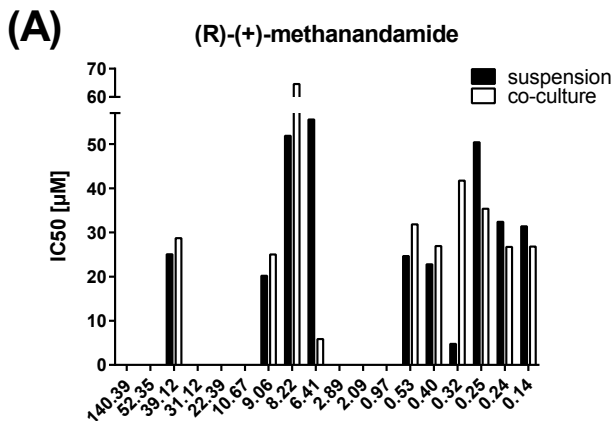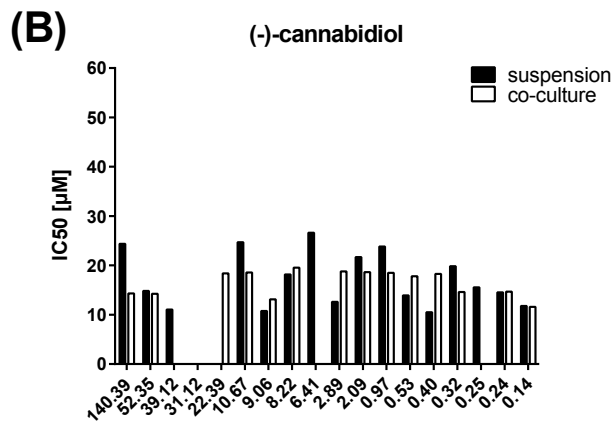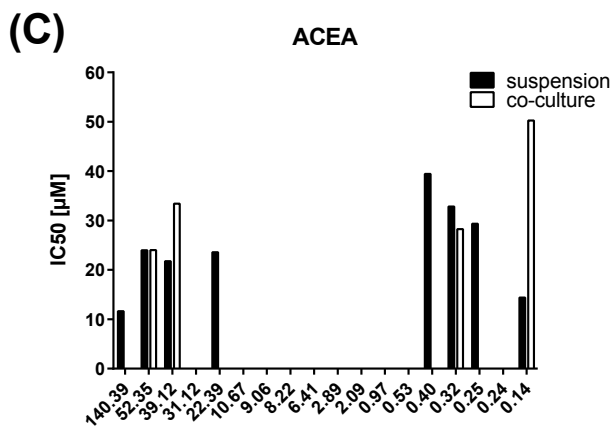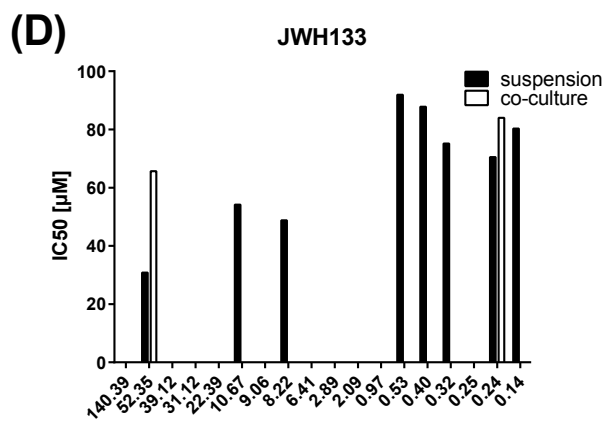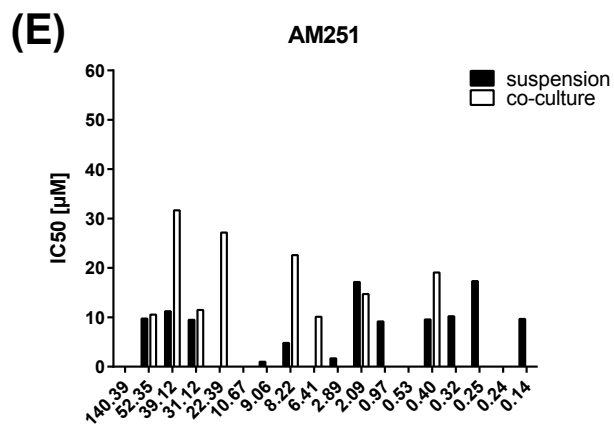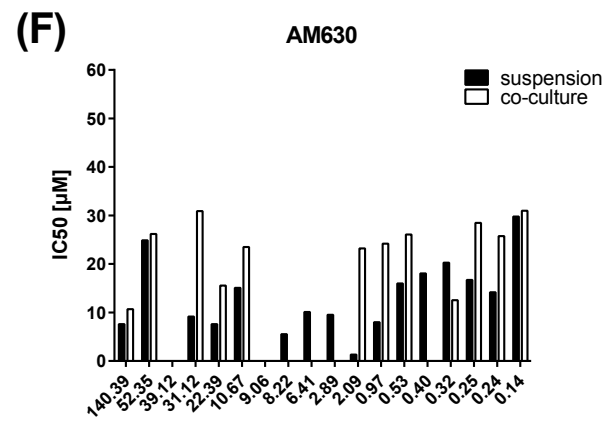

Supplement: S4 Fig — PBMC from CLL patients were incubated in triplicates in increasing compound concentrations in suspension and co-culture with M2-10B4 mouse fibroblast cells for 48h before viability was measured. (A) (R)-(+)-methanandamide (N = 10). (B) (-)-cannabidiol (N = 18). (C) ACEA (N = 16). (D) JWH133 (N = 16). (E) AM251 (N = 16). (F) AM630 (N = 16). The x-axis shows the measured mRNA expression for each CLL sample tested (healthy CD19 sorted cells set as 1) from highest (left) to lowest (right) expression. Absent values may indicate that i) sample was not tested, or ii) IC50 could not be calculated, or iii) 50% viability reduction could not be achieved. Note different scales on Y-axis for A and D. (PDF) [file pone.0156693.s004.pdf]

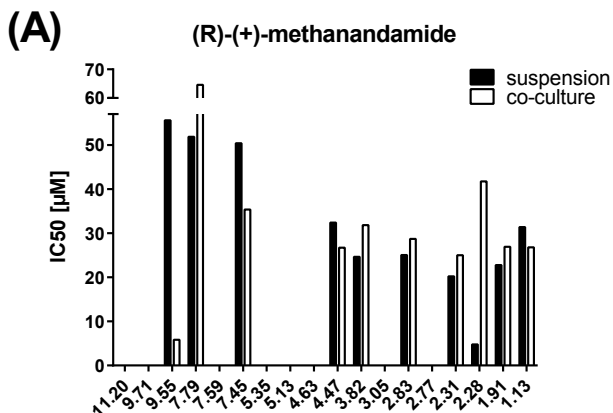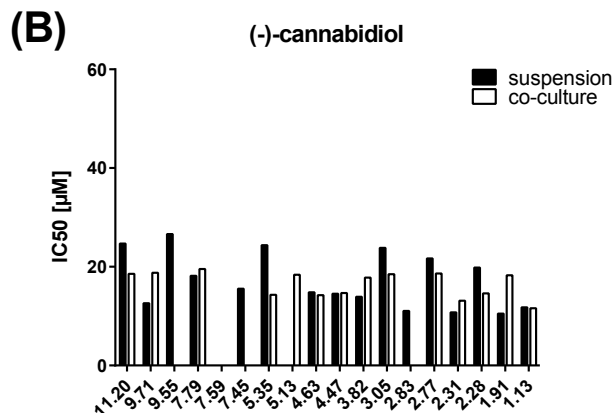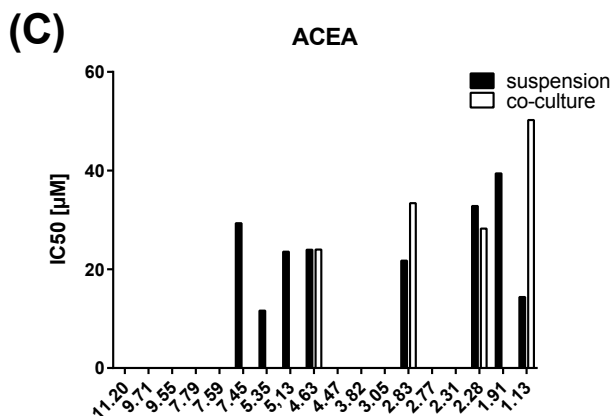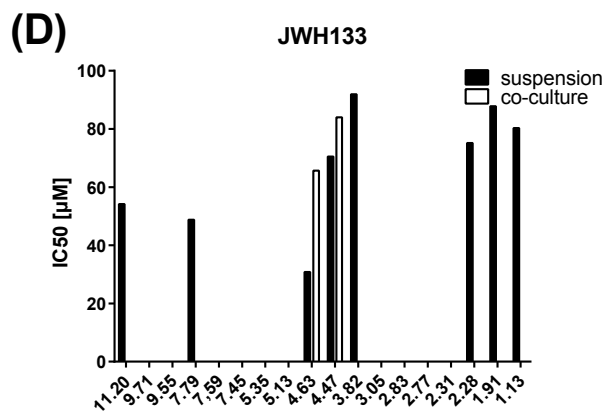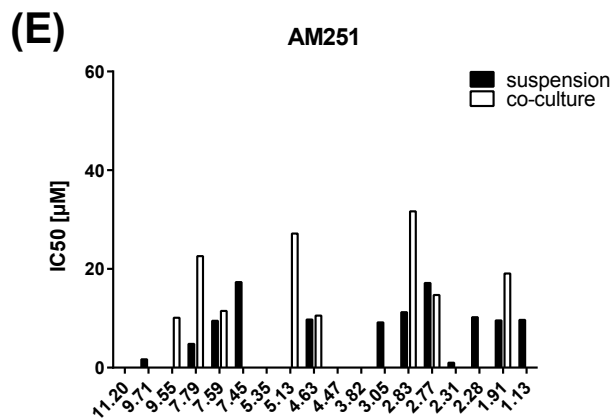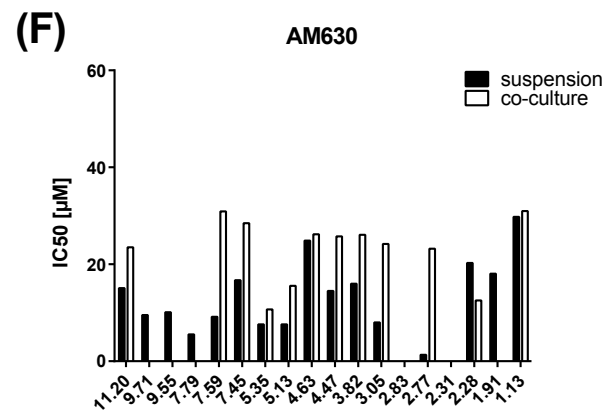

Supplement: S5 Fig — PBMC from CLL patients were incubated in triplicates in increasing compound concentrations in suspension and co-culture with M2-10B4 mouse fibroblast cells for 48h before viability was measured. (A) (R)-(+)-methanandamide (N = 10). (B) (-)-cannabidiol (N = 18). (C) ACEA (N = 16). (D) JWH133 (N = 16). (E) AM251 (N = 16). (F) AM630 (N = 16). The x-axis shows the measured mRNA expression for each CLL sample tested (healthy CD19 sorted cells set as 1) from highest (left) to lowest (right) expression. Absent values may indicate that i) sample was not tested, or ii) IC50 could not be calculated, or iii) 50% viability reduction could not be achieved. Note different scales on Y-axis for A and D. (PDF) [file pone.0156693.s005.pdf]

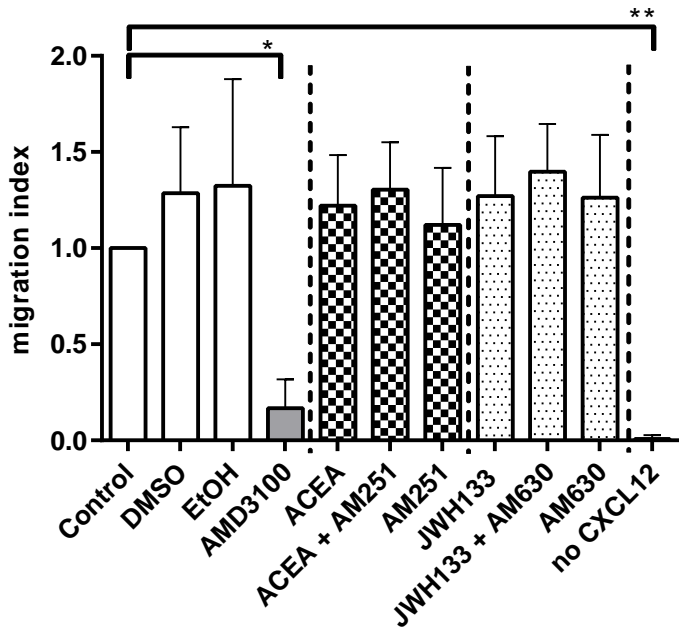

Supplement: S6 Fig — Primary cells of 5 CLL patients were pre-incubated with cannabinoids before being transferred to transwell plates and incubated for 4h for migration. Control experiments included CXCL12 alone (control), no CXCL12 (control w/o CXCL12), incubation with vehicle (DMSO, ethanol), and incubation with the CXCR4 inhibitor AMD3100. CLL cells were incubated either with agonist (ACEA, JWH133) or antagonist (AM251, AM630) before migration. In addition, cells were treated with antagonist before agonist incubation before migration was allowed (CB1: AMS251&ACEA; CB2: AM630&JWH133). Bars represent mean values of migration indices + standard deviations, hatched lines indicate experimental blocks.* p = 0.0016; ** p<0.0001. (PDF) [file pone.0156693.s006.pdf]
