# Supplementary material for: Cannabinoid Receptors Are Overexpressed in CLL but of Limited Potential for Therapeutic Exploitation
Source: PLoS One. 2016 Jun 1;11(6):e0156693. doi: 10.1371/journal.pone.0156693 (PMC4889125; doi:10.1371/journal.pone.0156693)
Supplement: S1 Table — (PDF) [file pone.0156693.s007.pdf]

**S1 Table. Clinical characteristics of the patients used in cannabinoid receptor mRNA analysis.**

| | |
|---|---|
| **Age at diagnosis** (N=102) [years] | |
| Median (Range) | 62 (25-80) |
| **Sex** (N=102) [%] | |
| Female:Male | 40.2:59.8 |
| **Binet at diagnosis (N=99)** [%] | |
| A | 83.8 |
| B | 12.1 |
| C | 4.0 |
| **Mutational status*** (N=90) [%] | |
| Unmutated | 45.6 |
| Mutated | 54.4 |
| **CD38** (N=93) [%] | |
| Low < 30 | 67.7 |
| High ≥ 30 | 32.3 |
| Median (range) | 10 (0-91) |
| **Cytogenetic aberrations** (N=96) [%] | |
| Del13q | 52.1 |
| Del11q | 24.0 |
| Del17p | 8.3 |
| Tris12 | 11.5 |
| Rearr14q | 11.5 |

*Cut-off 98% germline homology. Abbreviations: Del, deletion; Tris, trisomy; Rearr, Rearrangement.
