# Supplementary material for: Cannabinoid Receptors Are Overexpressed in CLL but of Limited Potential for Therapeutic Exploitation
Source: PLoS One. 2016 Jun 1;11(6):e0156693. doi: 10.1371/journal.pone.0156693 (PMC4889125; doi:10.1371/journal.pone.0156693)
Supplement: S2 Table — (PDF) [file pone.0156693.s008.pdf]

S2 Table. Compound concentrations for CD19+ sorted and compound concentrations and duration of incubations of unsorted PBMC before initiation of migration experiments. Drug concentrations were chosen such that cytotoxic effects would be minimal. Incubations with vehicle and with CXCR4 inhibitor AMD3100 served as controls.

	Pre-Incubation			Main-Incubation		
**Nr.**	**Concentration (µM)**	**Compound**	**Incubation time (h)**	**Concentration (µM)**	**Compound**	**Incubation time (h)**
1	Negative Control					
2	629	AMD3100	0.5			
3				10	ACEA	1
4	0.1	AM251	0.5	10	ACEA	1
5	0.1	AM251	0.5			
6				10	JWH133	1
7	0.1	AM630	0.5	10	JWH133	1
8	0.1	AM630	0.5			
9				0.1%	DMSO	1
10				0.1%	Ethanol	1
