# Supplementary material for: Cannabinoid Receptors Are Overexpressed in CLL but of Limited Potential for Therapeutic Exploitation
Source: PLoS One. 2016 Jun 1;11(6):e0156693. doi: 10.1371/journal.pone.0156693 (PMC4889125; doi:10.1371/journal.pone.0156693)
Supplement: S3 Table — (PDF) [file pone.0156693.s009.pdf]

S3 Table. Comparison of patient characteristics between CNR2 high and low mRNA expressing groups.

	CNR2 low	CNR2 high	p-value
**Age at diagnosis** [years]			
Median (Range)	65 (39-85)	60 (25-80)	
**Sex** [%]			
Female:Male	45.1:54.9	35.3:64.7	
**Binet at diagnosis** [%]			
A	85.7	82.0	0.616
B/C	14.3	18.0	
**Mutational status*** [%]			
Unmutated	46.7	44.4	0.832
Mutated	53.3	55.6	
**Lymphocyte doubling time** [%]			
Low < 1 year	25.5	21.7	0.667
High ≥ 1 year	74.5	78.3	
**CD38** [%]			
Low < 30	68.8	66.7	0.830
High ≥ 30	31.3	33.3	
Median (range)	7.5 (0-85)	17.0 (0-91)	
**Del13q** [%]			
Unmutated < 5.0	54.3	42.0	0.226
Mutated ≥ 5.0	45.7	58.0	
**Del11q** [%]			
Unmutated < 8.6	80.4	72.0	0.333
Mutated ≥ 8.6	19.6	28.0	
**Del17p** [%]			
Unmutated < 10.2	93.5	90.0	0.538
Mutated ≥ 10.2	6.5	10.0	
**Tris12** [%]			
Unmutated < 3.7	91.3	86.0	0.415
Mutated ≥ 3.7	8.7	14.0	
**Rearr14q** [%]			
Unmutated < 3.0	84.8	92.0	0.267
Mutated ≥ 3.0	15.2	8.0	

*Cut-off 98% germline homology. Abbreviations: Del, deletion; Tris, trisomy; Rearr, rearrangement. P-values: Pearson Chi-Square.
